# Movement-related activity in the internal globus pallidus of the parkinsonian macaque

**DOI:** 10.1101/2024.08.29.610310

**Published:** 2024-08-30

**Authors:** Daisuke Kase, Andrew J. Zimnik, Yan Han, Devin R. Harsch, Sarah Bacha, Karin M. Cox, Andreea C. Bostan, R. Mark Richardson, Robert S. Turner

**Affiliations:** 1. Department of Neurobiology, Center for Neuroscience and The Center for the Neural Basis of Cognition, University of Pittsburgh, Pittsburgh, PA, USA; 2. Aligning Science Across Parkinson’s (ASAP) Collaborative Research Network, Chevy Chase, MD, USA; 3. Department of Neuroscience, Columbia University Medical Center, New York, NY; 4. Zuckerman Institute, Columbia University, New York, NY, USA; 5. Department of Neurosurgery, Massachusetts General Hospital and Harvard Medical School, Boston, MA, USA

## Abstract

Although the basal ganglia (BG) plays a central role in the motor symptoms of Parkinson’s disease, few studies have investigated the influence of parkinsonism on movement-related activity in the BG. Here, we studied the perimovement activity of neurons in globus pallidus internus (GPi) of non-human primates before and after the induction of parkinsonism by administration of 1-methyl-4-phenyl-1,2,3,6-tetrahydropyridine (MPTP). Neuronal responses were equally common in the parkinsonian brain as seen prior to MPTP and the distribution of different response types was largely unchanged. The slowing of behavioral reaction times and movement durations following the induction of parkinsonism was accompanied by a prolongation of the time interval between neuronal response onset and movement initiation. Neuronal responses were also reduced in magnitude and prolonged in duration after the induction of parkinsonism. Importantly, those two effects were more pronounced among decrease-type responses, and they persisted after controlling for MPTP-induced changes in the trial-by-trial timing of neuronal responses. Following MPTP The timing of neuronal responses also became uncoupled from the time of movement onset and more variable from trial-to-trial. Overall, the effects of MPTP on temporal features of neural responses correlated most consistently with the severity of parkinsonian motor impairments whereas the changes in response magnitude and duration were either anticorrelated with symptom severity or inconsistent. These findings point to a potential previously underappreciated role for abnormalities in the timing of GPi task-related activity in the generation of parkinsonian motor signs.

## INTRODUCTION

What abnormalities in brain physiology underpin the motor impairments of Parkinson’s disease (PD)? One key step toward answering that question came with the discovery that lesions or stimulation of the posterior globus pallidus internus (GPi, primary output nucleus for the skeletomotor basal ganglia (1)) reduce the cardinal motor signs of PD, particularly akinesia, bradykinesia, and rigidity (2–5). That observation, coupled with the fact that dopamine replacement medication is also highly effective against those symptoms (6, 7), positions the GPi as a key anatomic link in the pathophysiologic cascade from dopaminergic denervation to symptom manifestation. Substantial uncertainty persists, however, regarding the specific abnormalities in GPi physiology that lead to parkinsonian symptoms.

Most pathophysiologic models of parkinsonism focus on the potential roles of abnormal tonic activity in GPi neurons in subjects at rest. For example, the classical “rate model” hypothesizes that an elevated tonic firing rate of GPi neurons leads to excessive inhibition of GPi-recipient motor centers (8, 9). An alternate group of models predict that abnormal patterns of GPi activity (e.g., bursty rhythmic activity and increased synchronization of firing between neurons) play a key role in PD pathophysiology (10, 11). An unanswered question for these models is how the abnormalities in resting neuronal activity are translated into a disruption of phasic motor commands, and thereby, impairments in active movement. Abnormalities in motor command-related single-unit activity are indeed common in BG-recipient regions of the thalamus (12), midbrain (13–15) and motor cortex by way of thalamus (16–18). The pathophysiologic models cited above assume that the translation from abnormal tonic discharge to disrupted motor commands occurrs in BG-recipient thalamo-cortical or midbrain circuits, after the activity has been transmitted out of the BG (19–21).

A seldom considered alternative is that abnormalities in the dynamic perimovement changes in GPi activity contribute directly to the disruption of motor commands in parkinsonism. Perimovement modulations in neuronal activity are common in the GPi of neurologically-normal animals (22–26). Given that GPi projections have a strong inhibitory influence on their targets (27), abnormal perimovement activity in GPi could have a direct disruptive influence on the generation of motor commands in GPi-recipient thalamocortical and midbrain motor circuits. Surprisingly, only one known study describes the activity of GPi neurons around the time of active movement in parkinsonian animals. Leblois et al. (28) reported that induction of parkinsonism in non-human primates (NHPs) led to an overall increase in the responsiveness of GPi neurons to active movement, a relative reduction in the incidence of perimovement decreases in firing (compared to that of increases), and shifts in the timing and duration of perimovement activity. Of particular note, perimovement activity shifted to *earlier* than normal onset times relative to movement onset and, in a subset of animals, earlier onset times relative to the sensory go-cue. That result does not align well with the common observation that go-cue -to-movement onset delays (i.e., reaction times, *RTs*) are increased in parkinsonian subjects (29–31) or with the general idea that parkinsonian bradykinesis reflects a general slowing of brain processes (32, 33). Leblois et al. also found that the duration of GPi perimovement responses was markedly prolonged after the induction of parkinsonism.

Some of these observations (28) are consistent with previously-proposed pathophysiologic mechanisms. For example, the increased responsiveness of GPi neurons to active movement fits with the general idea that parkinsonism is associated with a breakdown in the functional segregation between circuits throughout the BG (i.e., reduced neuronal selectivity for specific sensory stimuli (34–37). Also, Leblois et al.’s observation that perimovement decreases in firing become less common in parkinsonism could be seen as consistent with Nambu et al.’s (38) dynamic version of the classical rate model, in which under-activation of the GPi-inhibiting direct pathway (the “pro-kinetic” pathway) and over-activation of GPi-exciting indirect pathways (“anti-kinetic” pathways) work together to yield deficient perimovement decreases in GPi activity and concommitent inadequate disinhibition of GPi-recipient motor circuits (39). No established model, however, predicts Leblois et al.’s finding of a shift in the latency of GPi perimovement activity to earlier than normal onset times.

Here, we sought to replicate and extend upon the results of Leblois et al. (28) with the specific aim to gain better understanding of how induction of parkinsonism changes the magnitude and timing of GPi perimovement activity. We used a choice RT reaching task, variations of which have been used in previous studies of parkinsonism in non-human primates (16, 18). During task performance, single-unit neuronal activity was sampled from the skeletomotor territory of the GPi before and after the induction of parkinsonism by intracarotid infusion of the dopamine-specific pro-toxin MPTP. The distribution of different types of perimovement changes in activity (i.e., increases vs. decreases) and detailed parameters (latency, magnitude and duration) of perimovement changes in activity were compared between pre- and post-MPTP states. We then used a trial-by-trial analysis of response timing and duration to disambiguate measures that are typically confounded in standard mean across-trials analyses. Our trial-by-trial analysis also provided new insights into the effects of parkinsonism on the trial-to-trial variability of response timing and the temporal linkage of neural responses to different task events.

Unilateral injection of MPTP into the right intra carotid artery successfully induced parkinsonism on the left side of the body. A large fraction of GPi neurons continued showing perimovement responses after the induction of parkinsonism. Trial-by-trial analysis of response showed the reduction in response magnitude and prolongation of response duration in the de-jittered response. These effects of MPTP were exaggerated in decrease-type responses. Trial-by-trial analysis also revealed the increased variability of response onset in each trial. Furthermore, we found that the tight temporal linkage of response to the movement onset before the induction of parkinsonism shifted to the linkage to the go-cue after the induction. These changes following the induction of parkinsonism may contribute to the pathophysiology of parkinsonian motor symptoms.

## MATERIALS AND METHODS

### Animals

The subjects were two adult macaque monkeys (*Macaca mulatta*: G, female 7.1kg, age 8–10 years; I, female 7.5kg; age 10–13 years). Some data collected from these animals prior to MPTP administration were used for a previous project (40). All aspects of animal care were in accordance with the National Institute of Health Guide for the Care and Use of Laboratory Animals (41), the Public Health Service Policy on the Humane Care and Use of Laboratory Animals, and the American Physiological Society’s Guiding Principles in the Care and Use of Animals. All experimental protocols were evaluated and approved by the Institutional Animal Care and Use Committee.

### Surgery

General surgical procedures have been described previously (42, 43). The chamber implantation surgery was performed under sterile conditions with ketamine induction followed by Isoflurane anesthesia. Vital signs (i.e. pulse rate, blood pressure, respiration, end-tidal pCO2, and EKG) were monitored continuously to ensure proper anesthesia. A cylindrical titanium recording chamber was affixed to the skull at stereotaxic coordinates to allow access to the right arm-related part of the GPi via a parasagittal approach. The chamber was oriented parallel to the sagittal plane at an angle of 32˚. The chambers and head stabilization devices were fastened to the skull via bone screws and methyl methacrylate polymer. Prophylactic antibiotics and analgesics were administered post-surgically.

On protocols.io, we have posted two step-by-step protocols relevant to the surgical procedures (craniotomy https://dx.doi.org/10.17504/protocols.io.n92ldmz48l5b/v1 and implantation of head fixation and recording chamber hardware https://dx.doi.org/10.17504/protocols.io.5jyl8p9yrg2w/v1).

### Behavioral task

The animals performed a choice RT reaching task that has been described previously (43, 44)(DOI: 10.5281/zenodo.11397910). In brief, the animal was seated in a monkey chair facing a vertical black panel on which two target LEDs that were positioned at shoulder height 7 cm to the left and right of midline. The animal began a trial by placing the left hand on a metal ‘home-position’ bar positioned at waist height to the left of the left hip. Upon completion of a random duration start-position hold period (SPHP, 2–10 sec, uniform distribution randomized trial-to-trial) one of the target LEDs was illuminated (“go-cue”, left or right LED selected pseudo-randomly). The animal was required to move its hand from the home-position to the illuminated target LED within a 1 sec response window relative to the onset of the go-cue, and hold at the target position for 0.5–1.0 sec (uniform distribution). A drop of pureed food reward was delivered at the completion of successful trials via a sipper tube and a computer-controlled peristaltic pump. A trial was marked as an error and reward was not delivered if the animal failed to complete the hold period, reached to the incorrect target, or if the 1 sec response window was exceeded. The animal was allowed to return its hand to the home-position and initiate the next trial with no time restrictions. The presence of the hand at the home-position and at targets was detected by infrared proximity sensors (Takex, GS20N).

Some timing parameters of the task were adjusted following MPTP administration to make it easier for the parkinsonian animals to perform the task successfully. The maximum response window was expanded to 3 sec relative to onset of the go-cue, and the hold period at the target position was shortened to 0.1 sec.

In addition, parkinsonian animals often failed to return the affected left hand to the start position at the end of a trial, after reward delivery. A milder form of this phenomenon has been described previously(44, 45). In the present study, if an animal failed to initiate a return-to-home movement within 5 sec of reward delivery then the experimenter intervened and provided manual assistance to return the animal’s hand to the home-position. Both animals readily learned to accepted this manual assistance.

### Recording

The extracellular spiking activity of neurons in the GPi was collected using multiple glass-insulated tungsten electrodes (0.5–1.0 MΩ, Alpha Omega Co.) or 16-channel linear probes (0.2–1.0 MΩ, Plexon Inc.). Signals were amplified (4x), band-pass filtered (2 Hz – 7.5 kHz), digitized at 24 kHz (16-bit resolution: Tucker Davis Technologies), and saved to disk as continuous signals. When stable single-unit isolations were available in one or more channels, neuronal and behavioral data were collected while the animal performed the behavioral task.

On protocols.io, we have posted protocols relevant to electrophysiological recording (https://dx.doi.org/10.17504/protocols.io.bp2l6xx91lqe/v1) and recording chamber maintenance (https://dx.doi.org/10.17504/protocols.io.5jyl8ppm9g2w/v1).

### Administration of MPTP

After completion of observations in the normal state, a hemiparkinsonian syndrome was induced by injection of MPTP into the right internal carotid artery (ICA; 0.5 mg/kg, (46)). This model of parkinsonism was chosen to ensure that animals could maintain themselves and remain healthy for the months-long period of post-intoxication recording. Use of this model also increased the likelihood that animals would continue performing the operant task following intoxication (18). The ICA MPTP administration procedure was performed under general anesthesia (1–3% Isoflurane), and prophylactic antibiotics and analgesics were administered postsurgically.

We have posted on protocols.io the step-by-step protocol used to administer MPTP: (https://dx.doi.org/10.17504/protocols.io.dm6gp3zw5vzp/v1).

Animal G received one ICA infusion of 0.33 mg/kg MPTP hydrochloride (Sigma M0896–10MG). In animal I, stable parkinsonian signs appeared after two ICA infusions of MPTP (0.33 mg/kg) followed by four intra-muscular injections (0.33~0.55 mg/kg). The severity of parkinsonism was assessed by: (1) the degree of slowing and appearance of other impairments in the performance of the reaching task (see Results); (2) reduced overall mobility of the animal in its home cage accompanied by a dramatic increase in the tendency to turn toward the more depleted right hemisphere (clockwise)(46); (3) a marked reduction in the density of dopamine terminals in the dorsolateral putamen and substantia nigra compacta as measured by tyrosine hydroxylase (TH) staining in *post mortem* tissue. Dopamine replacement therapy was not administered at any time during the post-MPTP data collection period. Post-MPTP recording sessions started >30 days after the last MPTP administration.

### Histology

After the last recording session, each animal was given a lethal dose of sodium pentobarbital and perfused transcardially with saline followed by 10% formalin in phosphate buffer (for animal I) or 4% paraformaldehyde (for animal G). Both brains were cryoprotected with 20% Glycerol. The brains were blocked in place in the coronal plane, removed, cryoprotected with sucrose, and cut into 50 μm sections. Sections at 0.5 mm intervals were stained with cresyl violet for localization of microelectrode tracks. Selected sections were processed for immunohistochemistry to visualize tyrosine hydroxylase (TH) for documentation of the loss of dopaminergic cells in the substantia nigra pars compacta (SNc). We have posted on protocols.io the step-by-step protocol used for the immunohistochemical staining for TH (https://dx.doi.org/10.17504/protocols.io.4r3l2q1dql1y/v1).

### Analysis

#### Behavioral data

All error trials and outliers in task performance were excluded from further analyses. Error trials included reaches to the incorrect target and failures to reach the target within the allowed interval after the go cue (1 sec and 3 sec for pre- and post-MPTP respectively). Outlier trials were detected according to RT and movement duration. The RT was defined as the interval between the go-cue presentation and subsequent movement onset, and movement duration as the interval between movement onset and target capture. Trials were classified as outliers if either behavioral metric exceeded a threshold of 6 median absolute deviations from the mean.

#### Single-unit data

The stored continuous neurophysiologic signals were high pass filtered (Fpass: 300Hz, Matlab FIRPM, MathWorks, RRID: SCR_001622). For the bulk of these recordings, candidate action potentials were thresholded and sorted manually using Off-line Sorter (*OLS*, Plexon Inc., RRID: SCR_000012). Using that method, clusters of similar-shaped action potentials were identified across multi-way 2-D projections of waveform metrics. Clusters were accepted as representing the spiking of well-isolated single-units only if the cluster’s waveforms were of a consistent shape, could be separated reliably from other clusters as well as from background noise across a large fraction of the recording session, and violated a minimum refractory period criterion (1.5 ms) in only 0.5% of the resulting inter-spike intervals.

A subset of the neuronal data were sorted using a custom semi-automated spike-sorting algorithm (*TomSort*). The TomSort algorithm was used to identify single unit clusters in the neuronal recordings that were collected with 16-channel laminar probes in animal I following MPTP administration. After putative single unit clusters were identified by TomSort, those results were curated manually using OLS and the same acceptance criteria as applied when sorting was performed using OLS alone. A post-hoc comparison of results from the application of OLS and TomSort methods on the same recordings showed that TomSort identified nearly all of the single-units isolated using OLS alone. For example, across two recording sessions, TomSort successfully isolated 12 out of the 13 GPi units that were identified using OLS alone. But TomSort identified additional single-unit clusters that were not identified using OLS alone. Post-hoc inspection revealed that waveforms from those additional clusters were evident in the raw neuronal recordings, but those units were overlooked during manual sorting using OLS alone. The MATLAB code for TomSort is available on Github: (https://github.com/turner-lab-pitt/TomSort; DOI: 10.5281/zenodo.11176979).

We submitted all isolated units to a procedure that flagged instances of anomalous spike waveform features. Such instances may indicate, for example, that a cluster of waveforms did not originate in the soma of a GPi cell. Briefly, we summarized the median unit waveforms according to three features (47–50): the half-width, the difference in the peak and trough magnitudes (normalized by waveform amplitude), and the difference in the pre- and after-hyperpolarization peaks (normalized by their sum). For each MPTP state (and collapsed over the two subjects), we represented each unit as a point in a three-dimensional feature space, which was in turn submitted to the MATLAB implementation of DBSCAN (51). DBSCAN identifies outliers as points that lack neighbors that fall within a pre-defined distance. We discarded all outlier units, resulting in the exclusion of 9.40% and 3.17% of the units isolated pre- and post-MPTP, respectively. The MATLAB code for this analysis is available on Github (https://github.com/turner-lab-pitt/outlier-waveform-detection; 10.5281/zenodo.11118235), and input data for this code are available on Zenodo (DOI: 10.5281/zenodo.11077189).

After removing the units with outlier spike waveform features, we defined acceptable ranges of firing rates for pre- and post-MPTP populations separately. We calculated mean firing rates across whole recording periods for each unit population from both animals and then obtained the median and median absolute deviation (MAD) across those populations. The range of acceptable firing rates was defined as rates that fell within 1.5 × MAD of the median firing rate (22.9−117.7 Hz for pre-MPTP units and 1.5 −76.0 Hz for post-MPTP units). Single units were accepted for further analysis if their activity met the spike sorting, waveform shape and firing rate criteria described above and their activity was sampled over at least 10 trials for each target direction in the session.

##### Discharge rate and pattern at rest:

The effects of MPTP intoxication on resting neuronal activity were quantified using standard methods (52, 53). Those analyses were applied to spike trains during the start-position hold periods (SPHPs) of the behavioral task – intervals of unpredictable duration (2–10 sec) during which an animal held its left hand stationary at the start position while awaiting onset of the go-cue. A neuron’s mean firing rate was calculated as the total number of spikes detected across all hold periods divided by the summed duration of all hold periods. Episodes of burst firing – discrete period of markedly elevated firing rate – were detected using the Poisson Surprise Method (53, 54). Bursts were defined as groups of 4 or more spikes whose inter-spike intervals (ISIs) were unusually short compared with other ISIs of the same spike-train. We used a surprise threshold of 5, which equates to p<0.05 that the candidate burst would occur as a part of a Poisson-distributed sequence of spikes. The overall “burstiness” of a cell was quantified as the fraction of spikes that occurred during bursts relative to the total number of spikes found across all SPHPs.

Rhythmic modulations in firing rate were detected using a “shuffled normalization” method (55–57). The discrete Fourier transform (FFT) was applied to non-overlapping 512-ms long segments of a spike train’s delta function smoothed with a Hanning window of the same length. (These spike train segments were extracted separately from each SPHP to avoid possible anomalies due to data discontinuities at SPHP concatenation boundaries. SPHPs containing fewer than four spikes were excluded from this analysis.) The resulting “primary” spectral density estimate (0–500-Hz, 2-Hz resolution) was normalized by dividing it by a “control” spectrum. The control spectrum was the mean of 100 spectra computed after 100 shufflings of the same ISIs as used to compute the primary spectrum. This normalization compensated for distortions in spectral estimates attributable to a neuron’s refractory period and thereby improved the detection of low frequency oscillations (56). The shuffle-normalization procedure yielded spectra that varied around a normalized value of 1. Peaks in the normalized spectrum between 4-Hz and 100-Hz were tested for significance relative to the SD of the spectrum in the 150–250-Hz “control” range. The omnibus threshold for significance (p=0.05) included a Bonferroni correction for multiple comparisons (0.05 / 51 spectral points tested between 4-Hz and 100-Hz; actual threshold p<9.8×10^−4^). If a spectral peak exceeded the threshold at more than one frequency bin, then the central rhythmic frequency was defined as the spectral bin with the highest power.

##### Peri-movement discharge:

We tested for peri-movement changes in single-unit spike rate using a method described previously (43). First, spike density functions (SDFs) were constructed by convolving each unit’s spike time stamps (1 kHz resolution) with a Gaussian kernel (σ = 25 ms). Spike trains were aligned to the time of movement onset in each trial, and across-trial mean SDFs were constructed separately for reaches to each target. Significant modulations in the mean SDF were tested for during a test window that extended from the across-trials median time of go-cue onset to the across-trials median time of movement offset. The significance of modulations during that test window was determined relative to baseline defined as the mean and standard deviation of the mean SDF across a 700 ms window that ended at the beginning of the test window (defined above), after correcting for linear trends in the mean SDF over the baseline window. We defined a peri-event response as a statistically significant elevation or depression in the mean SDF that lasted at least 60 ms relative to baseline (e.g., Fig. 3A, solid vertical lines; t-test, one-sample versus baseline; omnibus p < 0.001 after Bonferroni correction for multiple comparisons). Significant peri-movement modulations in mean firing rate were classified as increase- or decrease-type “responses.” As described previously (58), the peri-movement modulation of some single-units was polyphasic, being composed of one or more significant increase and decrease in firing rate during the test window. Those responses were classified according to the sign of the earliest significant modulation – either polyphasic increase/decrease or polyphasic decrease/increase [referred to hereafter as “poly (+/−)” and “poly (−/+),” respectively].

#### Quantification of response metrics

The onset latency of a response was defined as the time of the earliest significant point in the response (1 ms resolution) relative to the alignment event of the mean SDF (Fig. 4A *vertical green line*). For monophasic increase- and decrease-type responses, the magnitude of a response was defined as the firing rate maximum or minimum, respectively, relative to the unit’s baseline firing rate estimated at the detected time of response onset (Fig. 4A). The duration of a response was defined as the full-width of the response at half of its maximum change in firing rate [i.e., full-width at half-max (*FWHM*), or at half-minimum for decrease-type responses]. For polyphasic responses, response magnitude and duration metrics were computed as outlined above, but only for the initial phase of the response.

#### Trial-by-trial detection of response onsets

Measures of response magnitude and duration taken from a mean across-trials SDF are susceptible to distortions that depend on how variable the timing of the response is from trial to trial (i.e., its “temporal jitter”). For example, in a comparison of across-trials mean SDFs, what appears to be reduction in response magnitude and increase in duration could in fact be attributable to a simple increase in the trial-to-trial variability of response timing (schematized in Fig. 4E-F). In other words, when measurements are taken from a mean across-trials SDF, the net effect of increased trial-to-trial jitter in response timing is indistinguishable from a true reduction in response magnitude and increase in response duration.

To resolve this ambiguity, we detected the onsets of neuronal responses on a trial-by-trial basis using a general approach that has been described in detail elsewhere (58, 59). First, only units found to have a significant perimovement response in the mean across-trials SDF were subjected to this analysis. Among those units, the class of response detected in the mean SDF was used to guide the subsequent trial-by-trial analysis. As an example, the approach used for monophasic perimovement increases is described here. Second, perimovement SDFs for individual trials were computed by convolving spike time stamps (1 ms resolution) with a Gaussian kernel (σ = 25 ms). Third, for each single trial SDF, we prepared a 200-point (200 ms) response kernel consisting of a simple step function in which the initial 100 points were set to the minimum firing rate in the single-trial SDF and the second 100 points are set to the maximum firing rate. Fourth, the quality of fit between the kernel and segments of the single-trial SDF were computed while shifting the kernel at millisecond steps from the time of go-cue onset to the end of movement. The time shift that resulted in the best fit (minimum summed squared error) between kernel and segment of single-trial SDF was taken to be the time of response onset for that single trial. This procedure was repeated for the single-trial SDFs from each valid behavioral trial. For decrease-type responses, we first inverted the single-trial SDF and then processed as described above for increase-type responses.

We then used those trial-by-trial measures of response timing to: 1) analyze the temporal linkage of responses to different task events (approach described in Results), 2) estimate the influence of parkinsonism on the fundamental variability of response timing, and 3) test for effects of parkinsonism on response metrics (magnitude and duration) after controlling for potentially confounding influences of differences in a response’s trial-to-trial temporal jitter.

For item 2) above, the fundamental variability of response timing was quantified as the residual trial-to-trial variability in response timing after controlling for potential influences of RT variability and the temporal linkage of the response to specific task events. First, the linear relationship between trial-by-trial response latency and RTs was determined by linear regression (Matlab, FITLM) to model the RT-dependent variability in response timing. Residuals from that regression were taken to reflect the fundamental trial-to-trial variability in response timing. The interquartile range (IQR) of those residuals was used as a measure of the trial-to-trial dispersion in response timing.

We also used the trial-by-trial estimates of response timing to compare response metrics (magnitude and duration) after controlling for the potentially confounding influences of differences in response temporal jitter. For each response, we removed the effects of temporal jitter by realigning single-trial SDFs to the response itself. First, in individual single-trial SDFs, we found the earliest maximum change in firing rate (Matlab, FINDPEAKS; firing rate maximum for increase-type responses and minimum for decrease-type responses) that occurred following the previously determined time of response onset for that trial. Second, the whole single-trial SDFs were shifted in time so that the times of peak change aligned across trials. Third, those realigned single-trial SDFs were averaged across-trials to yield a response-aligned mean SDF. Finally, response metrics (magnitude and duration) were extracted from the resulting response-aligned mean SDF using methods described above under *Quantification of response metrics*.

#### Statistics

##### ANOVA:

We tested for potentially interacting effects of parkinsonism and response type (e.g., increase versus decrease in firing rate) using two-way analyses of variance (MatLab, ANOVAN). Tukey’s test was used for the post-hoc analysis.

##### Standardized residuals analysis:

When results from a chi-square test rejected the null hypothesis, we isolated the source of the statistically significant effect using a standardized residuals approach (60, 61).

##### Interquartile range:

The dispersion of response onset times was quantified using the interquartile range (*IQR*), which is defined as the range that encompasses the central 50 percent of the data (62).

##### Clustering analysis:

Distributions were tested for universus poly-modal shapes using the Bayesian information criterion (*BIC*). BICs were obtained by fitting Gaussian mixture models to a distribution, considering a range of cluster numbers from one to four in this study (MatLab, FITGMDIST). The optimal number of clusters was determined by selecting the number of clusters with the smallest BIC (63).

## RESULTS

### Effects of MPTP administration: behavior and histology

The animals were rendered moderately hemiparkinsonian by the administration of MPTP as evidenced by changes in home cage behavior that were consistent with parkinsonism. Overall mobility was reduced, movement was slowed, and the animals adopted a hunched posture with increased flexion of the left limbs (contralateral to the depleted hemisphere). The animals showed a marked reduction in preference for using the left hand (e.g., for food retrieval), and an increased tendency to turn clockwise (toward the more-depleted right hemisphere)(46). Rigidity was increased at joints of the left arm and leg. Examinations performed at regular intervals throughout the post-MPTP data collection period supported the persistence of these signs. We found no evidence of tremor in either animal, consistent with previous observations that MPTP-treated macaques seldom exhibit frank parkinsonian tremor (46).

**Figure 1:**
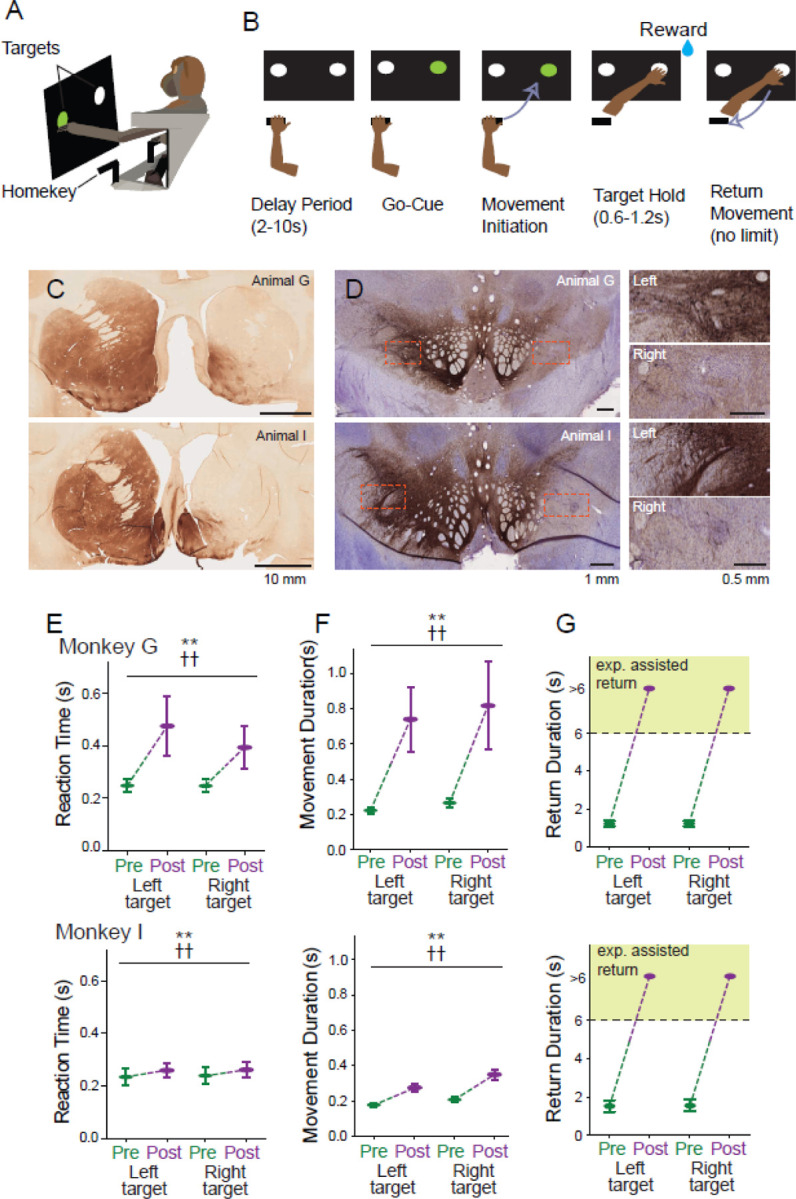
A-B. Schematic illustration of the recording chamber and the choice reaction time reaching task. C. Immunolabeling for tyrosine hydroxylase (TH) revealed the depletion of dopaminergic terminals in the right striatum of both animal G (top) and I (bottom). D. Immunolabeling for TH (brown) and Nissl staining (magenta) showed the absence of TH labeling in the right SNc (left) in both animal G (top) and I (bottom). Note that the TH labeling in the right ventral tegmental area was relatively preserved following the ICA MPTP administration. High-magnification details show the depletion of TH-labeled cells in the right SNc (right). Magenta: Nissl staining. Behavioral performance of both animals during the task, presented in a highly stereotyped fashion with short reaction time (E), movement duration (F), and return duration (G) pre-MPTP (green). E. Reaction time was significantly longer in both animals post-MPTP (magenta). F. Movement duration was significantly longer in both animals post-MPTP (magenta). G. Both animals displayed a severe impairment in the ability to return the hand to the start position (magenta). Statistical differences were computed using the 2-way ANOVA followed by Tukey’s test; (** p<0.001 for main effect of MPTP, †† p<0.001 for main effect of reach direction).

Despite the presence of these parkinsonian impairments, both animals were able to perform the choice RT reaching task, albeit at reduced levels of performance. Both animals showed significant slowing of RTs and of the durations of outward reaching movements to capture the target (Fig. 1E-G; 2-way ANOVA main effect of MPTP; F_(_1_,35652)_=31802, p < 0.01 for RT and F_(1,34986)_=50748, p < 0.01 for movement duration ). Although significant slowing was observed in both animals, the degree of slowing was much more severe in animal G than in animal I. In addition, both animals displayed a severe impairment in the ability to return the hand to the start position after reward delivery. In the neurologically normal state, prior to MPTP administration, animals returned their left hand to the start position with minimal delays. Following MPTP, however, the animals rarely if ever initiated a return movement. Instead, the animal’s left arm remained extended with the hand held stationary near the target for seconds following reward delivery. Similar freezing-like behavior has been described previously (44, 64). In order to collect adequate numbers of trials during post-MPTP recording sessions, if an animal did not return its hand to the start position within 5 seconds of reward delivery the experimenter intervened and manually moved the animal’s left arm to bring the hand into contact with the start position. Thus, in our post-collection analysis of behavior, any return movements that lasted longer than 6 sec were classified as “experimenter assisted return” (Fig. 1G) and considered to be categorically different from the rapid animal-initiated return movements of the pre-MPTP state. Experimenter-imposed manual assistance was required in essentially all of the trials collected following MPTP administration (chi-square test; *χ^2^*(1, n=37045) = 35743, p=0.0).

The loss of dopaminergic neurons from the substantia nigra pars compacta was quantified post mortem using TH immunohistochemistry (Fig. 1C and D).

### Neuronal database and activity at rest

**Figure 2:**
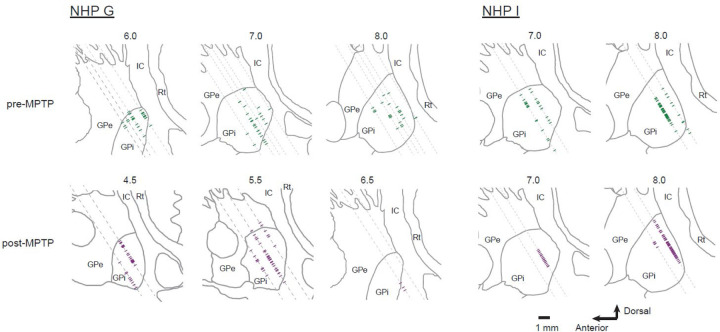
The locations of all GPi single-units included in the analysis are plotted on parasagittal sections at 1 mm intervals separately for animal G and I, pre- (green) and post-MPTP (magenta). The line drawing delineating the GPi boundary was taken from a standard atlas, which was subsequently adjusted to align with the structural MRIs and microelectrode mapping results from individual animals.

A total of 371 single-units met the criteria to be included as GPi neurons (180 and 191 pre- and post-MPTP respectively). For recording sessions before MPTP administration, units were studied over the course of 136 ± 92 trials of the behavioral task (mean ± SD; mean 68 trials for each of two movement directions). Following MPTP administration, units were studied over the course of 100 ± 42 trials of the behavioral task (mean ± SD; mean 49 trials for each of two movement directions). Units were sampled from relatively wide-spread regions of the posterior GPi during both pre- and post-MPTP sampling periods (Fig. 2).

The baseline activity of GPi neurons differed between pre- and post-MPTP periods in several ways, as would be expected with the induction of parkinsonism (10). Single-unit baseline firing properties were measured during a recording session’s start-position hold periods (SPHPs) – random duration (2–10 sec uniform distribution) periods of quiet attentive rest during which the animal held the left hand stationary at the start position in anticipation of go-cue presentation. Mean firing rates were reduced significantly post-MPTP (means±SEM: 69.0 ± 24.7 and 26.5 ± 19.2 Hz for pre- and post-MPTP respectively; Wilcoxon rank test; p<0.001, Table 1). Reductions in mean resting firing rates were found in both animals, but the magnitude of the reduction was much more dramatic in animal I (−50.9 sp/s) than in animal G (−31.4 sp/s; 2-way ANOVA MPTP × animal interaction; F_(1,367)_=19.7, p<0.01). That result runs contrary to predictions of the classical model of PD pathophysiology (8, 9). The spike-to-spike variability in firing was also elevated significantly post-MPTP (Wilcoxon rank test; p<0.001, Table 1).

Previous studies have also reported an increased tendency for GPi units to emit spikes within bursts (discrete periods of elevated firing rate) in parkinsonian animals (65, 66). In our dataset, the proportion of spikes that appeared within bursts increased markedly following MPTP administration (Wilcoxon rank test; p<0.05, Table 1). Both animals showed similar results when considered individually. Mean firing rates within bursts were reduced post-MPTP (means 296 and 122 spikes/sec pre- and post-MPTP, respectively; Wilcoxon rank test; p<0.01, Table 1). That result, however, was likely biased by the overall reduction in mean baseline firing rates post-MPTP (described above). When we normalized each unit’s intra-burst firing rates relative to its baseline firing rate, the mean normalized values increased significantly between pre- and post-MPTP periods (Wilcoxon rank test; p<0.05, Table 1).

Another often-observed neural correlate of parkinsonism in primates (human and non-human) is an increase in the prevalence of rhythmic modulations in firing rate (“oscillatory activity”) across a range of low frequencies (8–30 Hz; alpha and beta frequency bands) (67, 68). We used a shuffled normalization spectral analysis (56) to detect oscillatory spiking activity during SPHPs. The incidence of spectral peaks in the beta frequency range increased significantly following the induction of parkinsonism (standardized residual analysis; p<0.001, Table 1). Similar increases were found in the data from each animal considered individually, although that change reached significance only in NHP I (standardized residual analysis; p<0.001 and p=0.11 for NHPs I and G respectively). The increase in beta band oscillatory activity was accompanied by a significant reduction in the prevalence of spectral peaks in the gamma frequency range (standardized residual analysis; p<0.001).

To summarize, several aspects of GPi baseline activity changed following the administration of MPTP. The observed increases in burst firing and beta frequency oscillatory activity were both consistent with a large literature describing neurophysiologic hallmarks of parkinsonism (10, 69). Although the observed reduction in mean firing rates runs contrary to the classical model of parkinson’s pathophysiology (8, 9), two factors should be kept in mind. First, nearly all past studies examined the effects of parkinsonism on GPi activity in animals that were not engaged in a trained behavioral task. Here, we studied GPi activity during the SPHPs of a well-learned behavioral task. GPi activity during those two distinct behavioral states may be affected differently by the induction of parkinsonism. Second, several studies have reported that the mean firing rate of GPi neurons is not a reliable predictor of the severity of parkinsonian symptoms (65, 69, 70). Overall, the changes in GPi baseline activity described here following MPTP administration were consistent with the animals’ parkinsonian status.

**Figure 3:**
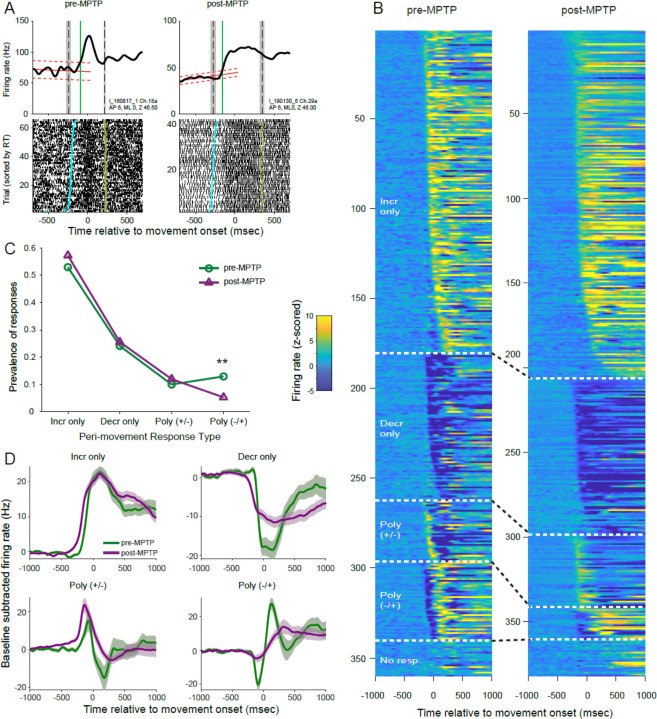
A. Activity of exemplar single-unit pre-MPTP (left) and port-MPTP (right) aligned to the time of movement onset (time zero). Vertical black dashed line and gray box indicate the median and IQR of the times of go-cue and the end of movement relative to movement onset. Horizontal red line represents the unit’s mean baseline activity ± SD. B. Spike-density functions of all single-units studied, sorted according to response form and response onset latency (earliest onset at the top for each response form) Left panel shows pre-MPTP data, and right panel shows post-MPTP data. Spike-density functions were z-scored relative to the mean baseline activity prior to go-cue presentation and displayed on a color scale. C. Overall proportions of peri-movement responses classified into the four response forms were slightly different between pre- and post-MPTP (p<0.01, chi-square test). The proportion of polyphasic (−/+) response was significantly smaller post-MPTP compared to pre-MPTP (** p<0.01, standardized residual analysis). D. Population-averaged spike-density functions for subpopulations with different response forms as labeled. Shaded area above and below the mean reflect the SEM.

Unit activity during SPHPs also frequently showed small but significant ramp-like changes in firing rate (Fig. 3A; linear regression; p<0.05). This observation, described previously for neurologically normal animals (40), is likely related to the progressively increasing probability (i.e., the hazard rate) of go-cue onset when, as in our task, SPHP durations are distributed uniformly (71, 72). Ramping activities were found in large fractions of the GPi units sampled both before and following MPTP administration (89% and 94% of units pre- and post-MPTP, respectively; Table 1). Positive and negative slopes were equally common among the ramp-like changes detected and that was equally true in both pre- and post-MPTP populations (chi-square test;*χ^2^*(3, n=371) = 4.08, p=0.13; Table 1). Our method for detecting the onset of perimovement changes in activity relative to a SPHP baseline took into account the presence of these ramp-like changes in firing rate.

### Perimovement responses persist post-MPTP

Next, we examined how perimovement modulations in firing rate were altered following the induction of parkinsonism. Figure 3A shows the activity of two example single units sampled from nearby sites in the GPi of animal I, one from the neurologically-normal animal and the other following MPTP administration (left and right subpanels, respectively). Both units showed a monophasic increase in firing that began during the reaction time interval [*vertical green line* denotes the estimated time of response onset relative to the unit’s baseline activity; i.e., linear trend ±confidence interval (CI) of activity prior to go-cue presentation, *red sloped lines* in Fig. 3A].

Nearly all single-units showed a significant perimovement change in discharge for at least one direction of movement. Contrary to previous report (28), the prevalence of perimovement changes did not increase following MPTP administration (98% of neurons were responsive both pre- and post-MPTP; chi-square test; *χ^2^*(1, n=371) = 0.213, p=0.64).

The form and timing of neuronal responses differed widely between neurons (Fig. 3B). First, we considered neuronal responses independently for each movement direction and tested for changes in the incidence of different forms of perimovement activity between pre- and post-MPTP populations (Fig. 3B). Monophasic changes in firing (composed of a simple increase or decrease in firing) were the most common, amounting to 79% and 83% of all responses under pre- and post-MPTP conditions, respectively. Monophasic responses became slightly more common following MPTP (chi-square test; *χ^2^*(1, n=689) = 2.49, p=0.11). Conversely, polyphasic responses – a series of one or more increase *and* decrease in firing – became less common following MPTP (21% pre-MPTP versus 17% post-MPTP). Polyphasic responses were subdivided further according to the sign of the initial change: increase followed by decrease [ *Poly(+/−)* ] and decrease followed by increase [ *Poly(−/+)* ]. All detected responses are shown sorted by response type and latency in Figure 3B.

Considering all four categories of response type, the prevalence of different response types changed with the induction of parkinsonism (chi-square test; *χ^2^*(3, n=689) = 11.30, p=0.010; Fig. 3C). That effect was attributable to an isolated reduction in the prevalence of Poly(−/+) type responses following MPTP administration (13% of responses pre-MPTP versus 5% post-MPTP; standardized residual analysis; p < 0.01). That reduction was accompanied by slight increases in prevalence of the other three response types following MPTP.

Thus, we found no evidence that parkinsonism was accompanied by a change in the overall prevalence of perimovement modulations in activity. We did find a modest reduction in the incidence of polyphasic responses that began with a decrease in firing.

### Perimovement decreases: preserved prevalence but reduced magnitude

Previous studies have suggested that parkinsonian motor symptoms arise from a selective loss of GPi perimovement decreases in firing (28, 39). In the current dataset, however, decreases in activity were equally common in the populations collected before and after MPTP administration. As a fraction of all monophasic changes in firing, decreases were equally common in the pre- and post-MPTP populations (31%; chi-square test; *χ^2^*(1, n=559) = 0.001, p=0.97). Furthermore, when the individual phases of all detected responses were considered independently, decreases in firing were also equally common in pre- and post-MPTP populations (chi-square test; *χ^2^*(1, n=820) = 0.105, p=0.75).

Inspection of the population averaged SDFs can provide insights to the features of responses that changed with the induction of parkinsonism (Fig. 3D). Population responses diverged from baseline firing at earlier latencies preceding movement onset as compared with responses pre-MPTP. That shift to an earlier latency was equally evident for Incr-only, Decr-only and Poly(+/−) response types, but barely perceptible for the Poly(−/+) type. Post-MPTP population responses also tended to have prolonged durations and reduced magnitudes as compared with responses pre-MPTP. Those reductions in response magnitude were most evident for decreases in firing rate (i.e., for the Decr-only response type and selectively for the decrease components of Poly(+/−) and Poly(−/+) response types).

### Perimovement responses are earlier, smaller and longer duration post-MPTP

**Figure 4:**
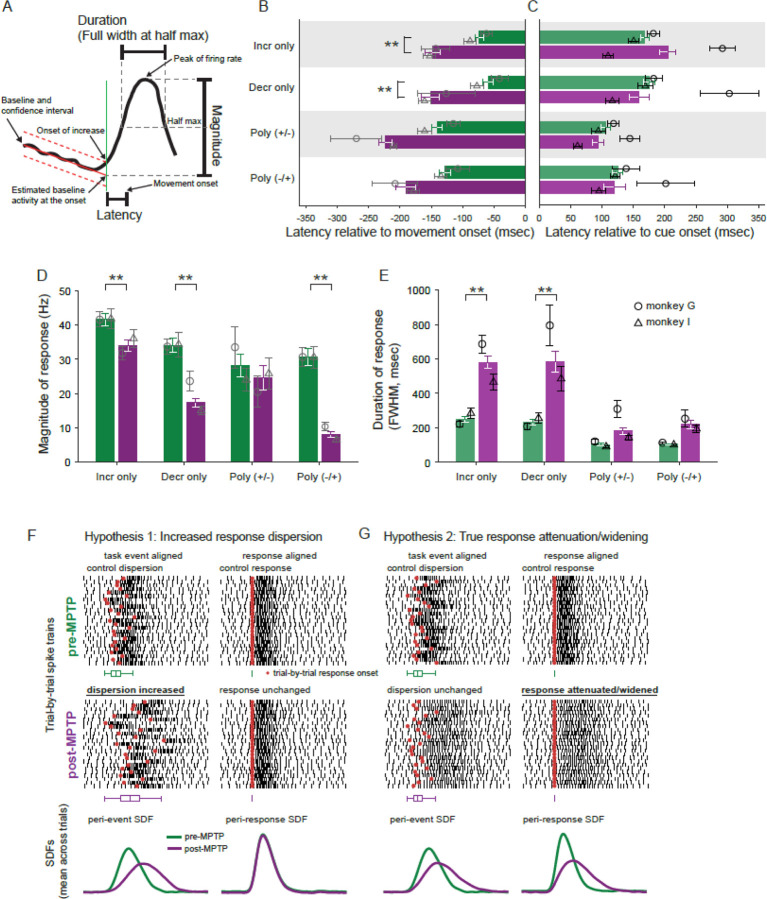
A. Schematic illustration depicting measures of response onset latency, response magnitude, and response duration. B and C. Response onset latencies relative to movement onset (B) and go-cue (C) for four subpopulations with different response forms (green: pre-MPTP, magenta: post-MPTP). MPTP administration significantly shifted the latencies relative to movement onset earlier (p<0.01, 2-way ANOVA main effect of MPTP; * p<0.05, ** p<0.01, Tukey’s test). D. Response magnitudes pre-MPTP (green) and post-MPTP (magenta). Magnitudes were reduced in size following MPTP administration (p<0.01, 2-way ANOVA main effect of MPTP; * p<0.05, ** p<0.01, Tukey’s test). E. Response duration pre-MPTP (green) and post-MPTP (magenta). Durations were prolonged following MPTP administration (p<0.01, 2-way ANOVA main effect of MPTP; ** p<0.01 Tukey’s test). F-G, Schematic illustrating potential mechanisms contributing to the reduction in mean response magnitude and increase in mean response duration. Top: spike rasters and bottom: spike-density function aligned at the task event (left) and trial-by-trial response onset (right). Red circles indicate trial-by-trial response onset. Box plots below the raster depict the distribution of trial-by-trial response onsets.

The results above suggest that parkinsonism was associated with changes in the timing and shape of perimovement modulations in GPi activity. To gain deeper insight into the specific response features affected, we took independent measurements of response onset latency, response magnitude (peak change from baseline) and response duration (full width at half max, FWHM) for perimovement modulations in the across-trials mean SDFs (see Fig. 4A). For polyphasic responses, those measures were obtained for the initial phase of the response. Measures were then compared between pre- and post-MPTP neural populations. In these analyses, responses for each direction of movement were considered separately.

Following MPTP administration, neuronal responses shifted to earlier onset latencies preceding movement initiation as compared with the onset latencies observed pre-MPTP (2-way ANOVA main effect of MPTP; F_(1,681)_=37.46, p < 0.01; Fig. 4B). Similar-sized shifts to earlier latencies were observed for all response types (2-way ANOVA MPTP × response-type interaction; F_(3,681)_=0.29, p=0.83) although post-hoc analysis indicated that that shift was significant only for Incr-only and Decr-only response types. In contrast, no significant change in latencies was found when response latencies were analyzed relative to the time of go-cue presentation (Fig. 4C).

Response magnitudes were reduced in size following MPTP (2-way ANOVA main effect of MPTP; F_(1,674)_=35.42, p<0.01; Fig. 4D). Although some reduction in response magnitude was observed for each response type, the reduction was more severe in decrease-type responses than in increase-type responses (2-way ANOVA MPTP × response-type interaction; F_(3,674)_=3.93 p<0.01). More specifically, monophasic decreases were reduced in size by 49% whereas monophasic increases were reduced in size by 19%. Similarly, among polyphasic responses, the initial decrease of Poly(−/+) responses saw a 74% reduction in magnitude whereas the initial increase of Poly(+/−) responses declined by only 16%.

Response durations (FWHM) were prolonged following MPTP (2-way ANOVA main effect of MPTP; F_(1,643)_=37.15, p<0.01; Fig. 4D). That prolongation was strikingly more severe for monophasic responses (233% and 252% for Incr- and Decr-only response-types, respectively; 2-way ANOVA MPTP × response-type interaction; F_(3,643)_=4.08, p<0.01) than for polyphasic responses (175% and 211% for Poly(+/−) and Poly(−/+) response-types, respectively). A similar pattern of effects on response magnitude and duration was found when those metrics were extracted from mean SDFs aligned relative to the time of go-cue presentation (supplementary Fig. S1 A-B).

The alterations in perimovement activity described thus far were taken from across-trials mean SDFs. Those measures, however, are susceptible to distortions that depend on how variable the timing of a response is from trial-to-trial (i.e., its temporal jitter) relative to the task event used to align the across-trials average. Two distinct mechanisms could account for a reduction in mean response magnitude and an increase in mean response duration: 1) an increased dispersion in the trial-to-trial timing of the response, but no change in the true magnitude or duration of the response itself (illustrated in schematic form in Fig. 4F, *Hypothesis 1*); or 2) the response itself is reduced in magnitude and prolonged in duration, while the trial-to-trial jitter in response timing is unchanged (Fig. 4G, *Hypothesis 2*). In other words, when measurements are taken from mean across-trials SDFs, the net effect of an increase in the trial-to-trial jitter in response timing is indistinguishable from a true reduction in response magnitude and increase in response duration. To address that ambiguity, we sought to control for the trial-to-trial jitter in response timing by: 1) estimating the times of onset of neuronal responses on individual trials; 2) creating response-aligned mean SDFs; and 3) quantifying response magnitude and duration from those response-aligned mean SDFs.

### Trial-by-trial response timing relative to task events

**Figure 5:**
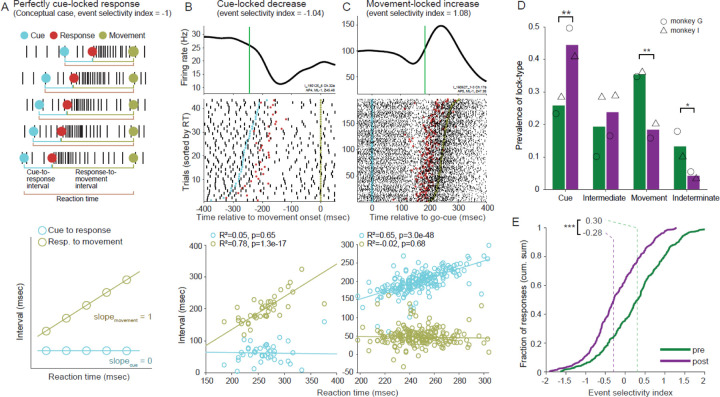
A. Top: schematic illustration of neural activity tightly linked to the go-cue. Cyan filled circle: go-cue, red filled circle: trial-by-trial response, filled yellow: movement onset. Bottom: scatter plot showing cue-to-response intervals (cyan open circle) and response-to-move intervals (yellow open circle). The cue-to-response intervals exhibit no correlation with reaction times, whereas response-to-movement intervals show a significant correlation. B-C. Activity of exemplar units locked to the cue (B) and movement onset (C), aligned to the time of movement onset. Top: trial-averaged spike-density function (black) and onset of change in firing rate (green). Middle: raster plot displaying trial-by-trial times of the go-cue (filled cyan circle), neural response (filled red circle), and movement onset (filled yellow circle). Bottom: relation between event-to-response intervals and reaction time (cyan open circle: cue-to-response intervals, yellow open circle: response-to-move intervals). The cue-locked unit (B) shows no correlation between cue-to-response intervals and reaction times, while the move-locked unit (C) shows no correlation between response-to-movement intervals and reaction time. D. Prevalence of units categorized by event locking activities pre- (green) and post-MPTP (magenta). The peak prevalence shifted from move-locked to cue-locked response following the induction of parkinsonism (p<0.001, chi-square test; * p<0.05, ** p<0.01, standardized residual analysis). Open circle: animal G, open triangle: animal I. E. Cumulative distributions of event selectivity index. The shift in event selectivity index from a positive to a negative value indicates the gradual shift of event-lock tendency rather than categorical shift as shown in panel D (p<0.001, Kolmogorov-Smirnov test).

The times of onset of neuronal responses on individual trials were detected using a technique similar to that described previously (58, 59)(see Materials and Methods). In addition to its utility for disambiguating response metrics, information on trial-to-trial response timing can provide valuable insights into the temporal (and, potentially, functional) linkages between neuronal responses and behavioral events. Inspection of the perimovement raster plots of many neurons suggested that responses on individual trials began at relatively fixed latencies either following the onset of the go-cue or relative to the time of movement onset (Fig. 5B and C, respectively). Timing relationships like these provide clues into the stage of processing a neural population participates in as part of a sensory-motor RT (e.g., in the task used here)(58, 73–75). That may include clues into the slowing of the RT process in parkinsonism.

Neural responses that participate at different stages of the sensory-motor RT process may be distinguished from each other by the variability in their timing relative to the appearance of the visual go-cue and the onset of movement. If a response has a tight temporal linkage to the sensory go-cue, then the time interval between the go-cue and the neuronal response should be constant regardless of the RT (i.e., slope ≈ 0, see schematic in Fig. 5A), whereas the interval between response onset and movement should covary strongly, across trials, with the behavioral RT (with slope ≈1). If, on the other hand, the response is linked tightly to movement initiation, then the interval between the go-cue and the response should covary with RT (e.g., Fig. 5C). Note that discrimination of those two temporal alignments depends critically on the presence of trial-to-trial variability in the behavioral RT (the interval between go-cue appearance and movement initiation).

To quantify those timing relationships, we calculated two separate linear regressions each comparing the trial-by-trial covariation of a time interval (go-cue–to–response or response–to–movement) with the behavioral RT. A significant positive relation (p<0.05) between response–to–move intervals and RTs, in the absence of a significant relation between cue–to–response intervals and RTs, was taken to indicate that the response was time locked to go-cue onset (i.e., *cue-locked*, Fig. 5A). Conversely, a significant positive relation between cue–to–response intervals and RTs, but not between response–to–move intervals and RTs, indicated that the response was time locked to movement onset (*move-locked*; Fig. 5C). When both regressions yielded significant positive slopes, the response was categorized as having “intermediate” locking a phenomenon also reported by DiCarlo et al. (75). If neither regression was significant, then the time locking was categorized as *indeterminate*.

Typical examples of single-unit responses time-locked to cue and movement are shown in Figure 5B and C, respectively. Movement-locked responses were the most common form of event locking in the pre-MPTP period (40.9% of responses; chi-square test; *χ^2^*(3, n=672) = 10.93, p<0.05, Fig. 5C), consistent with previous observations (58, 59, 76). Cue-locked responses were the second most common (23.0%). Intermediate-type locking (18.9%) and indeterminate event locking (17.2%) were relatively rare.

The prevalence of different event locking types changed markedly following the induction of parkinsonism (chi-square test; *χ^2^*(3, n=820) = 42.70, p<0.001; Fig. 5D). Movement-locking became far less common post-MPTP, dropping to 23.8% of responses, whereas cue-locking increased markedly to become the most common form of event locking (40.8% of responses). The prevalence of intermediate-type locking did not change significantly. Indeterminate locking became very rare in the post-MPTP population. Very similar changes in event locking were found when increase- and decrease-type responses were considered separately (see supplementary Fig. S2).

To gain deeper insight into the distribution of different forms of event locking, and the interacting effects of MPTP, we calculated an event locking index (*ELI*) that reflects the relative difference in slopes yielded by the two regressions (slope_cue-resp_ and slope_resp-mvt_) as follows: ELI = (slope_cue-resp_ − slope_resp-mvt_)/ (slope_cue-resp_ + slope_resp-mvt_). An ELI value ≤ −1 denotes perfect cue-locking, ELI ≥ +1 denotes perfect movement-locking, and an ELI value of 0 denotes a response whose timing covaries with the midpoint of the RT interval regardless of the overall duration of the RT.

Prior to MPTP administration, ELIs were spread across a broad Gaussian-like distribution centered on a median ELI of 0.30 (Fig. 5E). The smooth distribution provided no evidence for the presence of separable categories of event locking. Consistent with that conclusion, clustering analysis indicated that the distribution was most likely composed of one unimodal cluster (see Methods – Statistics; (63). These results imply that the discrete categories of event locking described above and previously (58, 59, 76) and Fig. 5D) are a product of the categorical analysis used. The broad smooth distribution of ELIs suggests that GPi responses participate instead at a multitude of steps in the sensory-to-motor processes that occur during the RT interval.

Following the induction of parkinsonism, the overall distribution of ELIs shifted markedly to the left, away from movement-locking and toward more cue-locked responses (median ELI = −0.29; Kolmogorov-Smirnov test; p<7.3 e^−12^; Fig. 5E). The shift was due in part to a selective reduction in responses closely locked to movement onset. Also evident, was a trend for the distribution of ELIs to skew further leftward post-MPTP (skew = 0.13 and 0.29 for pre- and post-MPTP, respectively), consistent with an increased representation of responses locked to cue-onset following the induction of parkinsonism.

To summarize, the normally strong temporal linkage of many GPi reponses to movement initiation (58, 59, 76) was reduced following the induction of parkinsonism. That was replaced by an increased temporal locking of responses to go-cue onset. That shift in coupling, combined with the prolongation of the response-to-movement interval described previously (Fig. 4B), may provide insights into the pathophysiology of increased RTs in parkinsonism (29, 77). Before discussing that idea in more depth, we examine the effects of parkinsonism on the fundamental variability in response timing and compare response metrics after controlling for the trial-to-trial jitter in response timing.

### Effects of MPTP on temporal dispersion of responses differed between animals

The trial-to-trial jitter in timing of neuronal responses became more variable following the induction of parkinsonism in one of the two animals. We measured the residual variability in response timing after regressing out the components of temporal variability attributable to differences in event locking and the trial-to-trial variability in RTs (see Methods – Analysis for details).

**Figure 6:**
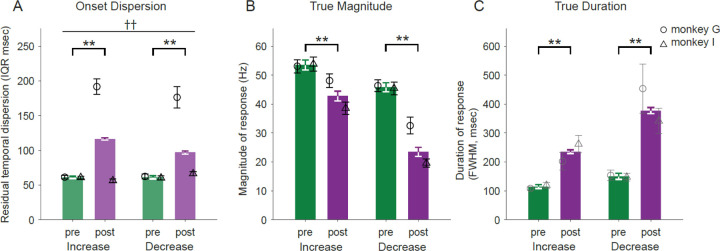
A. Summaries of residual temporal dispersion. B. Response magnitude from response-aligned spike-density function. C. Response duration from response-aligned spike-density function (green and magenta bars: pre- and post-MPTP respectively). Statistical differences for the residual temporal dispersion were computed using the 3-way ANOVA followed by Tukey’s test (†† p<0.01, MPTP × animal interaction; ** p<0.01, Tukey’s test). Statistical differences for the de-jittered responses magnitude and duration were computed using the 2-way ANOVA followed by Tukey’s test (** p<0.01). All values are presented as mean ± SEM. Open circle: animal G, open triangle: animal I.

Considering the mean across both animals, the temporal dispersion of responses increased significantly following MPTP (3-way ANOVA main effect of MPTP; F_(1,728)_=215.15, p<0.001; Fig. 6A). That result, however, was due completely to a marked effect of MPTP in neurons sampled from animal G (mean 213% and 183% increase in IQR for increase- and decrease-type responses, respectively) whereas minimal changes were found in animal I (mean 9% decrease and 10% increase in IQR for increase- and decrease-type responses, respectively; 3-way ANOVA MPTP × animal interaction; F_(1,728)_=224.54, p<0.001). The pattern of effects was very similar for increase- and decrease-type responses (3-way ANOVA main effect of response type; F_(1,728)_=0.03, p=0.85) and for responses categorized as cue-locked and movement-locked (see supplemental Fig. S3A and D). It is worth noting that the one animal that showed a large increase in the temporal dispersion of responses, animal G, was also the animal whose motor performance was more severely affected by MPTP (see Fig. 1E-F).

### De-jittered responses confirm smaller and wider responses post-MPTP

We suggested above that the MPTP-induced alterations in trial-averaged responses (i.e., reduced magnitude and prolonged duration; Fig. 4D, E) might be attributable to an underlying increase in the temporal jitter of response onset times across trials. That idea can now be addressed by using the trial-by-trial response onsets to re-align single trial SDFs (thereby removing the temporal jitter; see Methods – Analysis), average across trials and then calculate response metrics from the response-aligned SDFs. Polyphasic response types were excluded from this analysis due to the relatively small number of responses of those types.

Metrics taken from de-jittered responses showed effects that were relatively similar to those extracted from trial-averaged SDFs. De-jittered responses showed a reduction in magnitude following MPTP (2-way ANOVA main effect of MPTP; F_(1,706)_=97.77, p<0.001; Fig. 6B). Although both increase and decrease response types showed significant reduction, decrease-type responses showed a much stronger reduction in magnitude than increase-type responses (2-way ANOVA MPTP × response-type interaction; F_(1,706)_=10.24, p<0.01). Following the induction of parkinsonism, monophasic decreases were reduced in size by 48% whereas monophasic increases were reduced by 21%.

Similarly, the durations of de-jittered responses increased following MPTP (2-way ANOVA main effect of MPTP; F_(1,660)_=65.49, p<0.001; Fig. 6C). Notably, that prolongation was greater for decrease-type responses than for increase-type responses (106% and 152% for increase- and decrease-type responses, respectively; 2-way ANOVA MPTP × response-type interaction; F_(1,660)_=6.02, p<0.05). Note that this extra prolongation of decrease-type responses was not seen in metrics taken from trial-averaged SDFs (Fig. 4E) and thus was uncovered by the de-jittering process.

In conclusion, even after compensating for changes in the trial-to-trial jitter of response timing, response magnitudes were diminished and response durations were prolonged following the induction of parkinsonism. Of particular note, both of those effects of MPTP were exaggerated in decrease-type responses.

## DISCUSSION

We investigated the effects of MPTP-induced parkinsonism on the perimovement activity of neurons in the primate GPi. We found that parkinsonism was associated with a shift in the timing of perimovement responses to earlier onset times, reduced temporal linkage to movement and a weakening and prolongation of those changes. A particularly noteworthy result was that the weakening and prolongation of responses was more dramatic for decreases in firing as compared with those for increase-type responses. These results confirm and expand upon a previous description of parkinson’s-related abnormalities in GPi movement-related activity (28). Given that GPi neurons form the principal efferent pathway by which the BG influences motor control centers, these changes in GPi perimovement activity may be important factors in the pathophysiology of the motor symptoms of PD.

### Baseline activity in GPi

The mean firing rate of GPi neurons during periods of attentive rest was reduced following the induction of parkinsonism (Table 1). That result conflicts with predictions of the classical pathophysiologic model for PD (8, 9) and the many empirical observations that support that model (28, 66, 78–81). We, however, are not the first to find changes in resting activity that are at odds with the classical model (39, 53, 69, 82). How to explain these divergent findings is unclear at present. Potential explanations include differences between studies in the severity of parkinsonism induced, the specific MPTP intoxication protocol used, and, although less likely, other differences in the recording and signal processing techniques used. An additional potential explanation that applies to the present study in particular is that we measured mean firing rates while animals were engaged in a well-learned, attention-demanding behavioral task. Most previous studies recorded from animals that were not performing a defined behavioral task and, often in those studies, the animal’s behavioral state was not well controlled. Here, by measuring mean firing rates during the periods of attentive rest defined by the task’s start position hold intervals, we were able to assess the effects of parkinsonism on GPi activity during the same relatively well-controlled behavioral state before and after the induction of parkinsonism. The somewhat unexpected effect on mean firing rates found here may be related to the fact that our animals were engaged in a learned behavioral task combined with the possibility that the effects of parkinsonism on mean GPi firing rates differ between behavioral states.

In other respects, our results were consistent with previous descriptions of parkinsonism-related changes in GPi resting activity (Table 1). We found that the variability in inter-spike intervals and the mean proportion of spikes in bursts increased following the induction of parkinsonism, consistent with previous studies (53, 65, 66, 78, 83). In addition, the fraction of neurons with rhythmic modulations in firing in the beta frequency range increased with the induction of parkinsonism, in agreement with many previous studies (67, 68). Overall, the changes in GPi baseline activity found here were consistent with the animals’ parkinsonian status following MPTP administration.

### Preservation of perimovement activity post-MPTP

In many respects, the perimovement activity of GPi neurons was relatively unaffected by the induction of parkinsonism. The overall fraction of single units showing significant modulations in perimovement activity was high both before and following MPTP administration. Furthermore, the fundamental shapes of the responses detected and the rates at which those shapes appeared were roughly similar between pre- and post-MPTP conditions (Fig. 3C). Only the polyphasic(−/+) type, which accounted for a small fraction of responses pre-MPTP, became even less common following MPTP. These results differ from Leblois et al. (28) who reported that an increased fraction of neurons showed perimovement response following the induction of parkinsonism (49% and 67% pre- and post-MPTP, respectively). Leblois et al. also reported a dramatic decrease in the fraction of neurons showing perimovement decreases in firing (from 54% of neurons pre-MPTP to only 7% of neurons post-MPTP). Again, the reason for these differences between studies is uncertain, but may be attributable to differences in the animal model, severity of symptoms, or the methods used. Worth noting, however, is that we did find a differential diminution in the magnitude of decrease-type responses with the induction of parkinsonism, a result that will be discussed in detail below.

### Timing of GPi responses is uncoupled from movement initiation post-MPTP

Following the induction of parkinsonism, perimovement responses shifted to earlier mean onset times relative to movement initiation (Fig. 4B). In contrast, the latencies from go-cue delivery to mean response onset were minimally affected by parkinsonism. A similar pattern of results was described by Leblois et al. (28). We also found that the normally-common trial-to-trial linkage of GPi responses to movement onset was markedly reduced with the induction of parkinsonism (Fig. 5D-E). This second finding has not, to our knowledge, been describe previously.

These two results provide new insights into the neural mechanisms that lead in parkinsonism to the prolongation of the RT interval (i.e., the time interval between go-cue appearance and movement initiation) (29–31). Consistent with the animals’ parkinsonian status, we found that mean RTs increased significantly – markedly so in one animal – and the trial-to-trial variability in RTs increased as well (Fig. 1E). For each of these two effects on RTs, we defined a respective neural correlate: 1) the change in mean response latency relative to go-cue and movement and 2) the change in trial-to-trial locking relative to go-cue and movement. For both neural correlates, the change induced by parkinsonism was consistent with a breakdown in the temporal coupling of GPi responses to movement initiation.

The observation that latencies between go-cue delivery and response onset were minimally affected suggests that, in circuits upstream of the GPi, the *timing* of signal propagation is unchanged following the induction of parkinsonism. That view is consistent with a recent study of the effects of parkinsonism on the propagation of signals evoked by cortical stimulation via cortico-BG direct, indirect and hyperdirect pathways (39). In that study, parkinsonism strongly affected the magnitudes of signals propagated via different pathways, but the timing of those signals was unaffected. These results suggest that slowing in the conduction of neuronal signals, which is hypothesized to give rise to parkinsonian bradykinesia (32, 33, 84), is not present uniformly across all brain circuits. Moreover and perhaps surprisingly, slowed conduction is not apparent in the circuits immediately upstream of the GPi – circuits known to be affected strongly by MPTP-induced dopamine depletion.

We found that the time interval between neural response onset and movement initiation was both prolonged and more variable between trials following the administration of MPTP. One conceptually simple mechanism that could account for those effects is the presence, in circuits downstream of the GPi, of some form of pathology that causes a slowed and more variable conduction of the neural responses transmitted from the GPi. There is little evidence for frank pathology in the GPi itself in PD (85), aside from the loss of dopaminergic innervation (86, 87). A variety of disease-related alterations have been described for the thalamic and midbrain areas that receive monosynaptic projections from GPi (88–93). Pathologic changes have also been described for motor cortical areas, which are positioned two synapses downstream of GPi (94–96). Some combination of these pathologic changes may slow the downstream transmission of GPi task-related responses and, thereby, cause a prolongation of RTs. Of course this model assumes that responses transmitted from the GPi play a critical role in movement initiation.

An alternate idea is that abnormalities in the magnitude of GPi perimovement activity may play an active role in causing the increased delay to movement initiation and its increased variability. That idea is explored in more detail in the next section. A third possibility is that the prolongation of RTs in parkinsonism is attributable to slowed conduction along neural processing pathways that do not involve the GPi directly, but run in parallel to it.

Regardless of the underlying physiologic mechanism, our results indicate that the proximate cause for RT slowing lies either downstream of the GPi or along an independent parallel neural processing pathway. Recordings from regions directly downstream of the GPi should provide additional clarity regarding these potential mechanisms.

### Response magnitudes, especially of decreases, are attenuated post-MPTP

Perimovement changes in GPi activity were diminished in size and prolonged in duration following the induction of parkinsonism. Although reductions in magnitude were observed in both increase- and decrease-types of responses, perimovement decreases in firing were affected more severely. Monophasic decreases were reduced in magnitude by almost one half whereas monophasic increases were reduced by 19%, on average. Those effects persisted after controlling for the possibility that the observed attenuation and prolongation of mean responses could be explained by increases in the trial-to-trial jitter of response timing.

These results expand upon those from Leblois et al. (28) yet differ somewhat in detail. Leblois et al. report that perimovement decreases in firing became rare following the induction of parkinsonism. Decrease-type responses to proprioceptive perturbations showed a similar selective reduction in occurrence (28, 97). We did not observe a reduction in the rate of occurrence of decrease-type responses, but did find that perimovement decreases were attenuated in magnitude preferentially in comparison with the effects on perimovement increases. This disparity in results may be explained by differences between studies in the severity of symptoms, the task used or the analysis methods applied. Most obviously, what may be detected as a reduction in response magnitude in one study could easily appear as a reduction in occurrence in another depending on the study’s statistical power and analysis methods applied. Our results agree, however, with Leblois et al.’s overall conclusion that parkinsonism is associated with a differential attenuation of perimovement decreases in firing. Generalizing further beyond active movement, Chiken et al. (39) studied the effects of parkinsonism on responses evoked in GPi by electrical stimulation of sites in cortex. They found a selective attenuation of decrease-type responses – responses thought to be mediated via conduction of signals along the pro-kinetic cortex→striatum→GPi “direct” pathway (38). The direct pathway originates in striatal projection neurons that express the D1 dopamine receptor (D1-SPNs). Excitation of normally-functioning D1-SPNs produces a monosynaptic GABA-mediated inhibition of GPi neurons and thereby, transient decreases in GPi firing. Thus, a degradation of D1-SPN function would lead to a differential attenuation of GPi decreases in firing.

Several lines of evidence support the view that function of the direct pathway is selectively impaired in parkinsonism. D1-SPNs show marked reductions in spine density and dendritic complexity following chronic dopaminergic denervation (98, 99). Multiple studies report that dopamine denervation reduces the intrinsic excitability of D1-SPNs (reviewed in 100) although not all studies concur with that finding (98, 101). Finally, selective excitation of D1-SPNs restores motor function in a rodent model of PD (102) mediated by an apparent increase in the magnitude of pauses in firing of BG output neurons (103).

The present results have important implications for understanding the pathophysiology of PD. The canonical model of BG function states that, in the normal dopamine-replete brain, task-related reductions in GPi activity facilitate movement through disinhibition of neurons in BG-recipient regions of motor thalamus and midbrain (104, 105). Thus, in parkinsonism, a selective attenuation of decrease-type responses as seen here would result in deficient perimovement disinhibition of motor circuits and thereby hypokinetic/bradykinetic movements. According to this model, the slowing of movement seen in parkinsonism can be attributed to an attenuation of task-related decreases in GPi firing and their disinhibitory effect on downstream motor circuits.

An alternative model arises from the longstanding and well-supported idea that the BG plays a central role in procedural learning (106–108). In a key study, Yttri & Dudman (109) showed that selective excitation of D1-SPNs, which is known to increase the magnitude of pauses in BG output neurons (103), results in a prolonged increase in the rate of expression of the behavior that was being performed during that excitation. Those results suggest that decrease-type changes in GPi activity serve as a reinforcement learning signal in downstream brain areas. Thus in parkinsonism, a persistent attenuation of GPi task-related decreases, as observed here, may lead to impairments in motor performance through a process of repeated inadequate reinforcement, which could also be termed aberrant learning. Further credence for this general model of PD pathophysiology comes from the observation that parkinsonian impairments on motor tasks worsen the more the motor task is practiced (110–112).

We also observed an attenuation of task-related increases in firing. The two most prominent sources of net excitatory effects on GPi neurons are the glutamatergic inputs from the subthalamic nucleus and facilitation-through-disinhibition effects from the external pallidum. A weakening of task-related increases may be attributable to pathologic changes that have been described for both pathways (113–117). Though not as severe as the attenuation of decrease-type responses, this weakening of increase-type responses may also play a role in the pathophysiology of PD. The canonical model of BG function views task-related increases in GPi firing as a mechanism to inhibit potentially competing motor plans in BG recipient brain areas. Thus, attenuation of those increases in parkinsonism would result in inadequate inhibition of competing motor plans and contribute to the muddled selection and execution of appropriate motor plans.

We speculated in the section above that abnormalities in the magnitude of perimovement responses may play a direct role in the prolonged and more variable delay between GPi response onset and movement initiation. To elaborate, smaller than normal responses transmitted from parkinsonian GPi may disrupt the function of downstream motor control circuits and impair their ability to play-out motor commands in a timely and orderly fashion. In that way, the deficient GPi response magnitudes, which may arise from dysfunction in circuits upstream of the GPi (39), might also give rise to slowed initiation of movement (i.e., prolonged RTs). Studies of the effects of parkinsonism on perimovement activity in regions downstream of the GPi would provide a way to test that possibility.

### Responses durations, especially of decreases, are prolonged post-MPTP

Perimovement changes in activity were also prolonged in duration following the induction of parkinsonism. Similar to the observed effects on response magnitude, the durations of decrease-type responses were prolonged to a greater degree than those of increase-type responses (Fig. 6C; after controlling for the confounding effects of increased trial-to-trial jitter). This similarity suggests that the two effects, depressed response magnitude and prolonged duration, arise from similar pathophysiologic mechanisms. What those mechanisms are remains a matter of speculation, but may be related to some combination of the pathologic changes described above for direct, indirect, and hyperdirect pathways (98–100, 113–117). The consequences of prolonged response duration and relation to pathophysiology are also uncertain.

## Limitations

One inherent limitation of the present work is that the results are correlative. We cannot be certain whether the observed abnormalities in GPi responses cause changes in behavior, or reflect those changes, or are merely correlative. The fact that most GPi responses began during the RT interval places GPi responses at an appropriate timing to influence the initiation and execution of movement, but a causal role is by no means guaranteed. Experiments that perturb circuit function will be of assistance in clarifying the causal roles of the abnormalities described here.

It is important to acknowledge the simplistic nature of the concept, used widely above, of circuits “upstream” and “downstream” of the GPi. BG-thalamocortical circuits are organized as re-entrant loop circuits in which signals transmitted from the GPi can influence the very brain structures that send signals into the same BG circuit (118). Considering the typical transit times for these circuits (39, 40) and the durations of GPi responses, there is ample time during a GPi response for abnormal GPi output to loop back and influence the function of the “upstream” circuits that are generating that response. This re-entrant circuit organization may make it possible for the effects of even subtle dysfunctions of the circuit to be amplified as signals are passed several times through the circuit.

## Conclusions

In summary, remarkably few studies have examined the effects of parkinsonism on the perimovement activity of neurons in the GPi, the principal output nucleus of the primate BG. We found that perimovement responses were present in the parkinsonian brain at roughly the same overall abundance and distribution of different response types as seen in the neurologically normal state. Nonetheless, parkinsonism was associated with three principal abnormalities in perimovement activity: 1) The timing of GPi responses became uncoupled from movement onset both with respect to both mean latency and trial-to-trial variability in timing. 2) Response magnitudes were attenuated. 3) Response durations were prolonged. The effects on both response magnitude and duration were accentuated in decrease-type responses. These abnormalities in GPi perimovement responses may contribute to the pathophysiology of parkinsonian motor symptoms. In particular, the differential attenuation of decrease-type responses is consistent with the concept that decrease-type GPi responses are pro-kinetic such that their attenuation may play a key role in the pathophysiology of parkinsonian bradykinesia. Future studies of the effects of parkinsonism on perimovement activity in brain regions immediately downstream of the GPi are likely to clarify the pathophysiologic significance of the changes observed here.

## Supplementary Material

Supplement 1

## Figures and Tables

**Table 1:** 

	NHP G		NHP I		Total	
	pre-MPTP	post-MPTP	pre-MPTP	post-MPTP	pre-MPTP	post-MPTP
Number of Units	92	82	88	109	180	191
<Activity at rest>						
Firing rate (mean ± sd)	68.6 ± 22.8	37.2 ± 21.2	69.3 ± 26.6	18.4 ± 12.7	69.0 ± 24.7	26.5 ± 19.2
Coefficient of variation of ISI (mean ± sd)	1.1 ± 0.4	1.3 ± 0.5	1.1 ± 0.5	14 ± 0.7	1.1 ± 0.5	1.4 ± 0.6
Proportion of spikes in burst (mean ± sd)	0.10 ± 0.11	0.20 ± 0.17	0.11 ± 0.12	0.20 ± 0.20	0.10 ± 0.12	0.20 ± 0.19
Normalized Intra-burst FR (mean ± sd)	4.7 ± 1.3	6.0 ± 6.4	4.4 ± 1.5	6.8 ± 9.7	4.6 ± 1.4	6.5 ± 8.4
Oscillation at rest	47 (51%)	33 (40%)	28 (32%)	23 (21%)	75 (42%)	56 (29%)
Theta (4 – 7.9 Hz)	**0**	**3 (8%)**	0	1 (4%)	**0**	**4 (6%)**
Alpha (8 – 12 Hz)	**1 (2%)**	**4(11%)**	1 (3%)		2 (2%)	4 (6%)
Beta (12.5 – 30 Hz)	27 (49%)	24 (65%)	**5 (16%)**	**17 (65%)**	**32 (37%)**	**41 (65%)**
Low Beta (12.5 – 20 Hz)	0	23 (92%)	3 (60%)	5 (29%)	3 (9%)	28 (67%)
High Beta (20.5 – 30 Hz)	27 (100%)	2 (8%)	2 (40%)	12 (71%)	29 (91%)	14 (33%)
Gamma (30.5 – 100 Hz)	**27 (49%)**	**6 (16%)**	**25 (81%)**	**8 (31%)**	**52 (60%)**	**14 (22%)**
Ramping of firing rate	79 (86%)	74 (90%)	81 (92%)	106 (97%)	160 (89%)	180 (94%)
Positive slope	32 (41%)	33 (45%)	35 (43%)	50 (47%)	67 (42%)	83 (46%)
Negative slope	47 (59%)	41(55%)	46 (57%)	56 (53%)	93 (58%)	97 (54%)

Underlined: significant difference bewteen pre- and post-MPTP by Wilcoxon rank sum test

Bold: significant difference between pre- and post MPTP by residual analysis after chi-square test

## Data Availability

Both electrophysiological data and behavioral data are available on the DANDI Archive at DANDI:000947/0.240510.2211. All MatLab code and the processed source data sufficient to reproduce all figures and tables are in preparation and will be available on Zenodo.

## New & Noteworthy

Perimovement responses were present in the parkinsonian GPi at roughly the same overall abundance as seen in the neurologically normal state. Nonetheless, parkinsonism was associated with three abnormalities in perimovement activity: 1) Timings of GPi response became uncoupled from movement onset both with respect to both mean latency and trial-to-trial timing variability. 2) Response magnitudes were attenuated. 3) Response durations were prolonged. The effects on both response magnitude and duration were accentuated in decrease-type responses.
