# Supplementary material for: Movement-related activity in the internal globus pallidus of the parkinsonian macaque
Source: bioRxiv. 2025 May 30:2024.08.29.610310. Originally published 2024 Aug 30. Preprint. [Version 2] doi: 10.1101/2024.08.29.610310 (PMC11383679; doi:10.1101/2024.08.29.610310)
Supplement: Supplement 1 [file NIHPP2024.08.29.610310v2-supplement-1.pdf]

bioRxiv preprint doi: https://doi.org/10.1101/2024.08.29.610310; this version posted May 30, 2025. The copyright holder for this preprint (which was not certified by peer review) is the author/funder, who has granted bioRxiv a license to display the preprint in perpetuity. It is made available under aCC-BY 4.0 International license.

SUPPLEMENTAL FIGURE

Figure S1

A

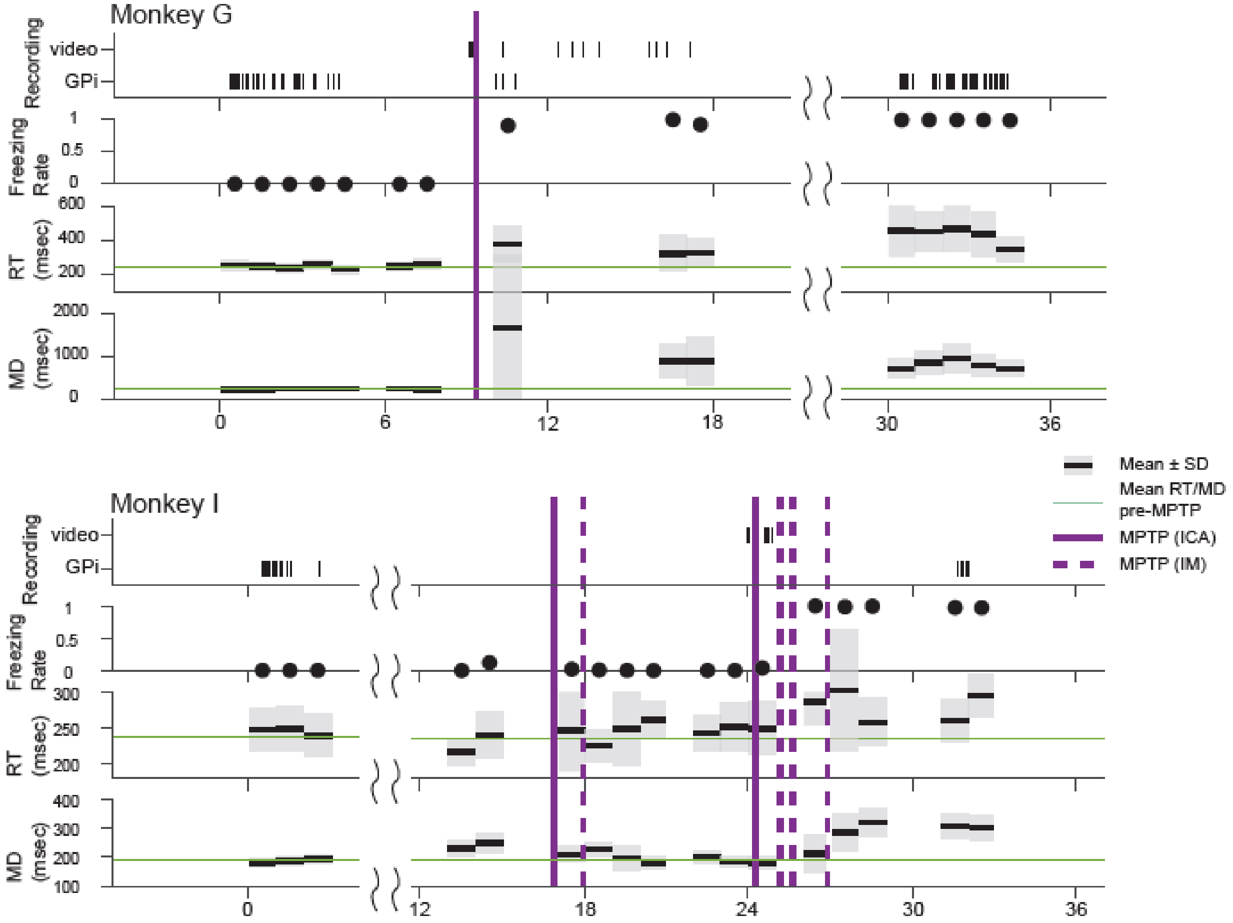

B

Monkey G

Days from 1st recording	Method	MPTP Dose
274 days	ICA	3.3 mg in 60 ml of saline (0.42 mg/kg)

Monkey I

Days from 1st recording	Method	MPTP Dose
499 days	ICA	2.5 mg in 60 ml of saline (0.33 mg/kg)
531 days	IM	2.6 mg (0.33 mg/kg)
716 days	ICA	2.6 mg in 60 ml of saline (0.33 mg/kg)
750 days	IM	3.9 mg (0.50 mg/kg)
764 days	IM	4.4 mg (0.55 mg/kg)
803 days	IM	4.4 mg (0.55 mg/kg)

bioRxiv preprint doi: https://doi.org/10.1101/2024.08.29.610310; this version posted May 30, 2025. The copyright holder for this preprint (which was not certified by peer review) is the author/funder, who has granted bioRxiv a license to display the preprint in perpetuity. It is made available under aCC-BY 4.0 International license.

Summary of the experimental timeline. The top and bottom panels correspond to monkeys G and I, respectively. In each panel, tick marks indicate the dates of recording sessions – video recordings of behavior in the home cage (*top of the top row*) and recordings of extracellular neural activity used in the current study (*bottom of the top row*). The rate of occurrence of freezing-like behaviors, reaction times, and movement durations are shown in respective rows second from top to bottom and plotted as means for each month data were collected. *Shading*: +/- SD for reaction time and movement duration. In reaction time and movement duration panels *horizontal green lines*: mean values across pre-MPTP sessions. *Vertical magenta lines*: times of MPTP administration via intra-carotid (*solid lines*) and IM (*dashed lines*) routes. Additional details on MPTP administration are provided below the timeline.

bioRxiv preprint doi: https://doi.org/10.1101/2024.08.29.610310; this version posted May 30, 2025. The copyright holder for this preprint (which was not certified by peer review) is the author/funder, who has granted bioRxiv a license to display the preprint in perpetuity. It is made available under aCC-BY 4.0 International license.

Figure S2

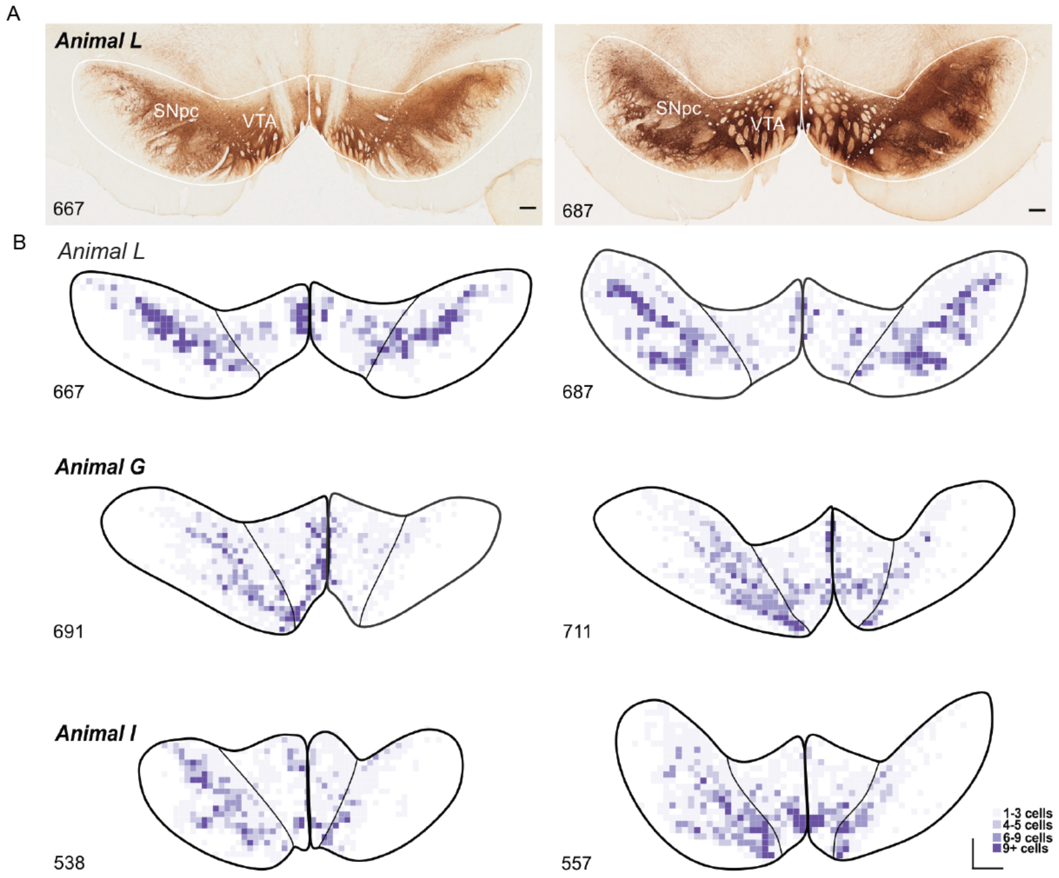

C

Animal L (Control)

Section ID		667 TH-positive neuron count	667 Area (mm²)	667 TH-positive neuron density (cell/mm²)	687 TH-positive neuron count	687 Area (mm²)	687 TH-positive neuron density (cell/mm²)	Total TH-positive neuron count	Total Area (mm²)	Total TH-positive neuron density (cell/mm²)
Left	SN	886	14.31	61.91	1138	17.04	66.78	2024	31.35	64.56
	VTA	395	5.46	72.34	255	5.94	42.93	650	11.40	57.20
Right	SN	652	14.31	45.56	1072	17.36	61.75	1724	31.67	54.44
	VTA	399	6.08	65.63	324	7.02	46.15	723	13.10	55.19
Total	SN	1538	28.62	53.74	2210	34.40	64.24	3748	63.02	**59.47**
	VTA	794	11.54	68.80	579	12.96	44.68	1373	24.50	**56.04**

Animal G (MPTP)

Section ID		691 TH-positive neuron count	691 Area (mm²)	691 TH-positive neuron density (cell/mm²)	711 TH-positive neuron count	711 Area (mm²)	711 TH-positive neuron density (cell/mm²)	Total TH-positive neuron count	Total Area (mm²)	Total TH-positive neuron density (cell/mm²)	TH-positive neuron density (% of control)
Left	SN	1051	24.56	42.79	1239	24.56	55.45	2290	49.12	46.62	78.39%
	VTA	653	9.04	72.23	513	8.61	59.58	1166	17.65	66.06	117.88%
Right	SN	128	15.36	8.33	389	17.55	22.17	517	32.91	15.71	**26.42%**
	VTA	412	9.83	41.91	479	7.63	62.78	891	17.46	51.03	91.06%

Animal I (MPTP)

Section ID		538 TH-positive neuron count	538 Area (mm²)	538 TH-positive neuron density (cell/mm²)	557 TH-positive neuron count	557 Area (mm²)	557 TH-positive neuron density (cell/mm²)	Total TH-positive neuron count	Total Area (mm²)	Total TH-positive neuron density (cell/mm²)	TH-positive neuron density (% of control)
Left	SN	931	16.99	54.80	1034	18.29	56.53	1965	35.28	55.70	93.66%
	VTA	282	6.68	42.22	418	5.77	72.44	700	12.45	56.22	100.32%
Right	SN	293	15.88	18.45	367	18.29	20.07	660	34.17	19.32	**32.49%**
	VTA	286	5.00	57.20	352	5.89	59.76	638	10.89	58.59	104.55%

bioRxiv preprint doi: https://doi.org/10.1101/2024.08.29.610310; this version posted May 30, 2025. The copyright holder for this preprint (which was not certified by peer review) is the author/funder, who has granted bioRxiv a license to display the preprint in perpetuity. It is made available under aCC-BY 4.0 International license.

Tyrosine hydroxylase (TH)-positive cells were depleted preferentially from the right substantia nigra (SN) following MPTP administration. *A*. Immunohistochemical labeling for TH in the healthy control animal L revealed dense populations of TH-positive somata in both the SN and ventral tegmental area (VTA). *B*. Density maps of TH-positive somata in the SN and VTA demonstrate a marked depletion from the right SN of animal G (middle) and I (bottom), with no such depletion observed in the healthy control animal L (top). Outlines show estimated borders between VTA and SNpc for two 50 um think sections spaced 1 mm apart through the midbrain of animals L, G and I. Color squares indicate the number of infected neurons in successive 200-um x 200-um bins (see color key). *C*. Summary of the number and density of TH-positive somata identified in the SN and VTA. TH-positive neurons were depleted markedly yet selectively from the right SN (bold text in far-right column) in both MPTP-treated animals.

bioRxiv preprint doi: https://doi.org/10.1101/2024.08.29.610310; this version posted May 30, 2025. The copyright holder for this preprint (which was not certified by peer review) is the author/funder, who has granted bioRxiv a license to display the preprint in perpetuity. It is made available under aCC-BY 4.0 International license.

Figure S3

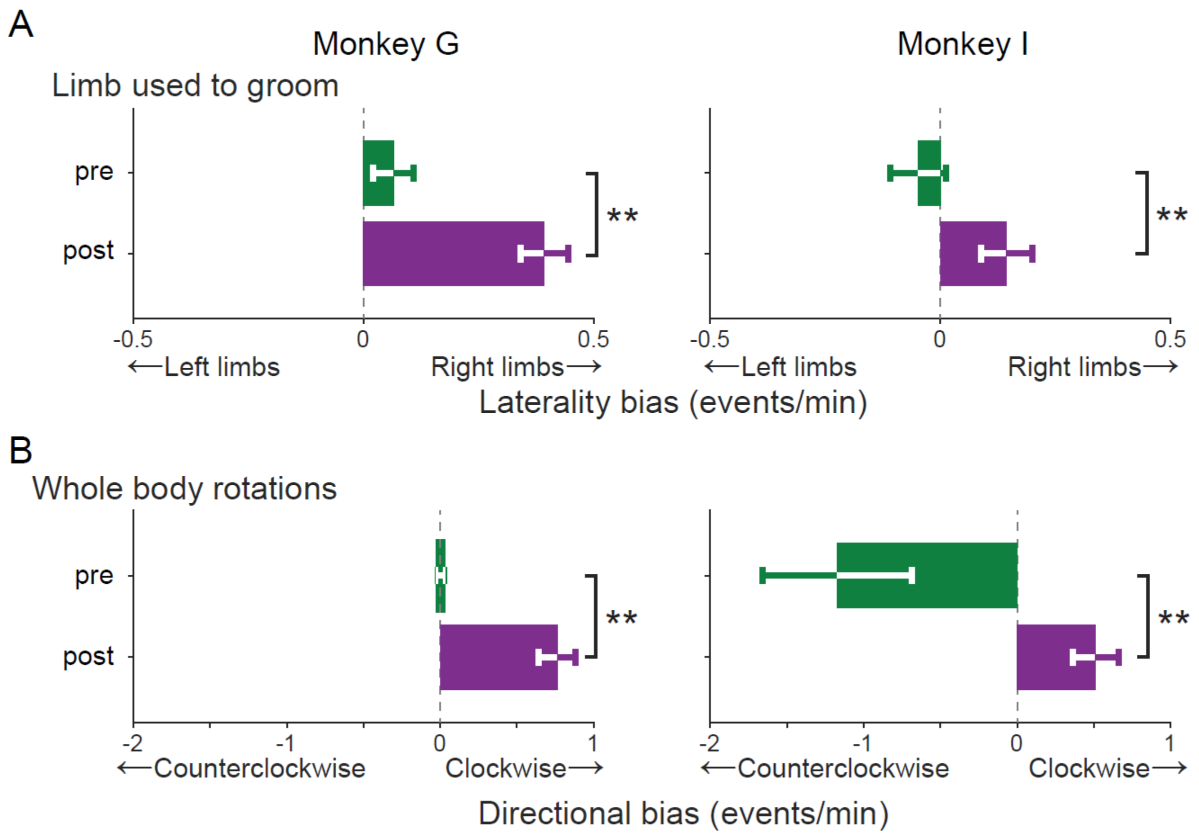

MPTP administration reduced use of the contralateral limbs and whole-body rotations toward the more affected side of the body. *A*. The rate of left limb use relative to right limb use during grooming behaviors before (green) and after (magenta) MPTP administration, shown separately for monkey G and I (left and right panels, respectively). *B*. The rate of clockwise whole-body rotations relative to that of counterclockwise rotations. Statistical comparisons were performed using the Wilcoxon rank-sum test (**$p < 0.01$).

bioRxiv preprint doi: https://doi.org/10.1101/2024.08.29.610310; this version posted May 30, 2025. The copyright holder for this preprint (which was not certified by peer review) is the author/funder, who has granted bioRxiv a license to display the preprint in perpetuity. It is made available under aCC-BY 4.0 International license.

Figure S4

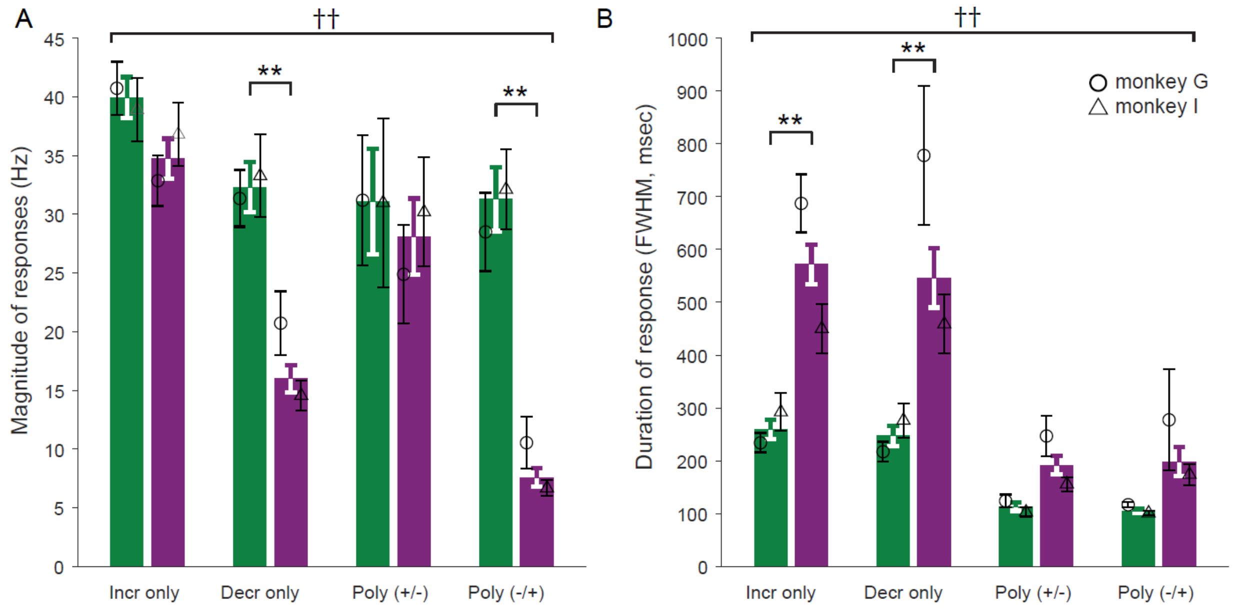

MPTP : F(1,668)=29.70, p=7.1e-08
Response : F(3,668)=19.57, p=3.6e-12
Monkey : F(1,668)=0.20, p=0.65
MPTP x Resp: F(3,668)=4.77, p=0.0027
MPTP x Monk: F(1,668)=0.29, p=0.59
Resp x Monk: F(3,668)=0.23, p=0.88

MPTP : F(1,633)=42.30, p=1.6e-10
Response : F(3,633)=16.98, p=1.3e-10
Monkey : F(1,633)=5.45, p=0.020
MPTP x Resp: F(3,633)=3.09, p=0.026
MPTP x Monk: F(1,633)=24.92, p=7.7e-07
Resp x Monk: F(3,633)=0.27, p=0.84

Effects of MPTP on response magnitude and duration as quantified from go-cue aligned SDFs. Results from this analysis were closely similar to those in measures taken from movement-aligned SDFs (Fig. 4D-E; following the same conventions). Results from 3-way ANOVA (MPTP × response type × animal) are shown below. *A*: The reduction in response magnitude was more severe in decrease-type responses (** p<0.01, Tukey's test). *B*: Response durations were prolonged following MPTP administration, and that prolongation was more prominent for monophasic responses (** p<0.01, Tukey's test).

bioRxiv preprint doi: https://doi.org/10.1101/2024.08.29.610310; this version posted May 30, 2025. The copyright holder for this preprint (which was not certified by peer review) is the author/funder, who has granted bioRxiv a license to display the preprint in perpetuity. It is made available under aCC-BY 4.0 International license.

Figure S5

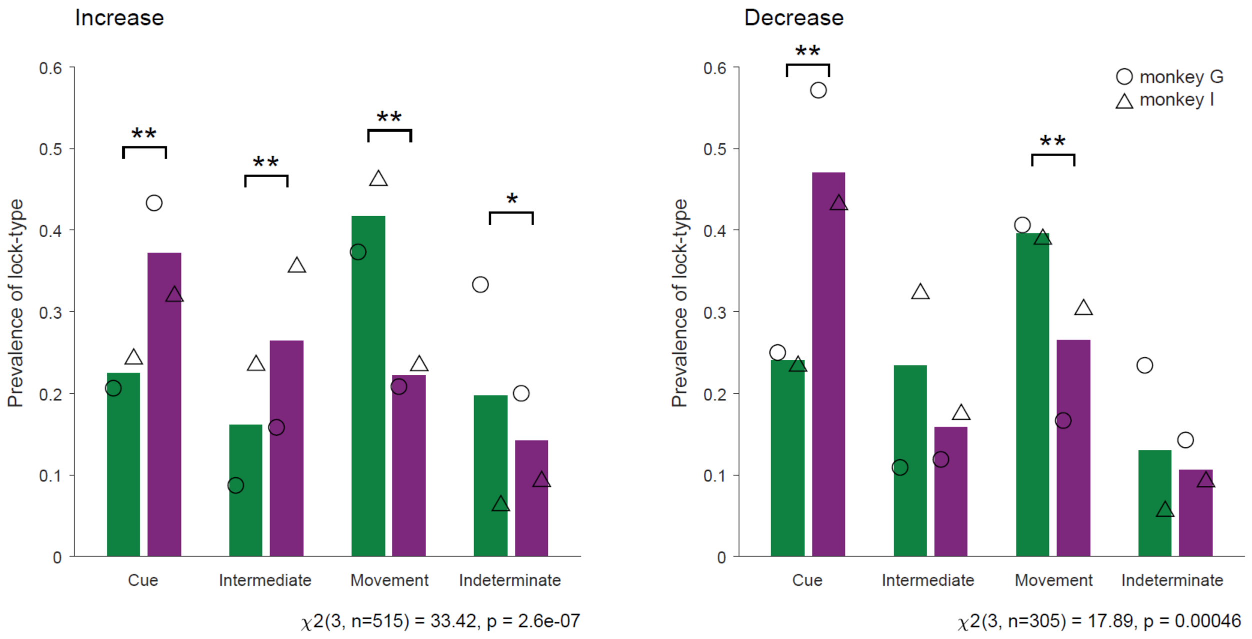

The increased prevalence of cue-locked responses following MPTP (i.e., Fig. 5D) was found in both increase- (*left*) and decrease-type response populations (*right*). Results from chi-square analysis are shown below figure panels (* $p<0.05$, ** $p<0.01$, adjusted residual analysis). *Open circles*: monkey G. *Open triangles*: monkey I

bioRxiv preprint doi: https://doi.org/10.1101/2024.08.29.610310; this version posted May 30, 2025. The copyright holder for this preprint (which was not certified by peer review) is the author/funder, who has granted bioRxiv a license to display the preprint in perpetuity. It is made available under aCC-BY 4.0 International license.

Figure S6

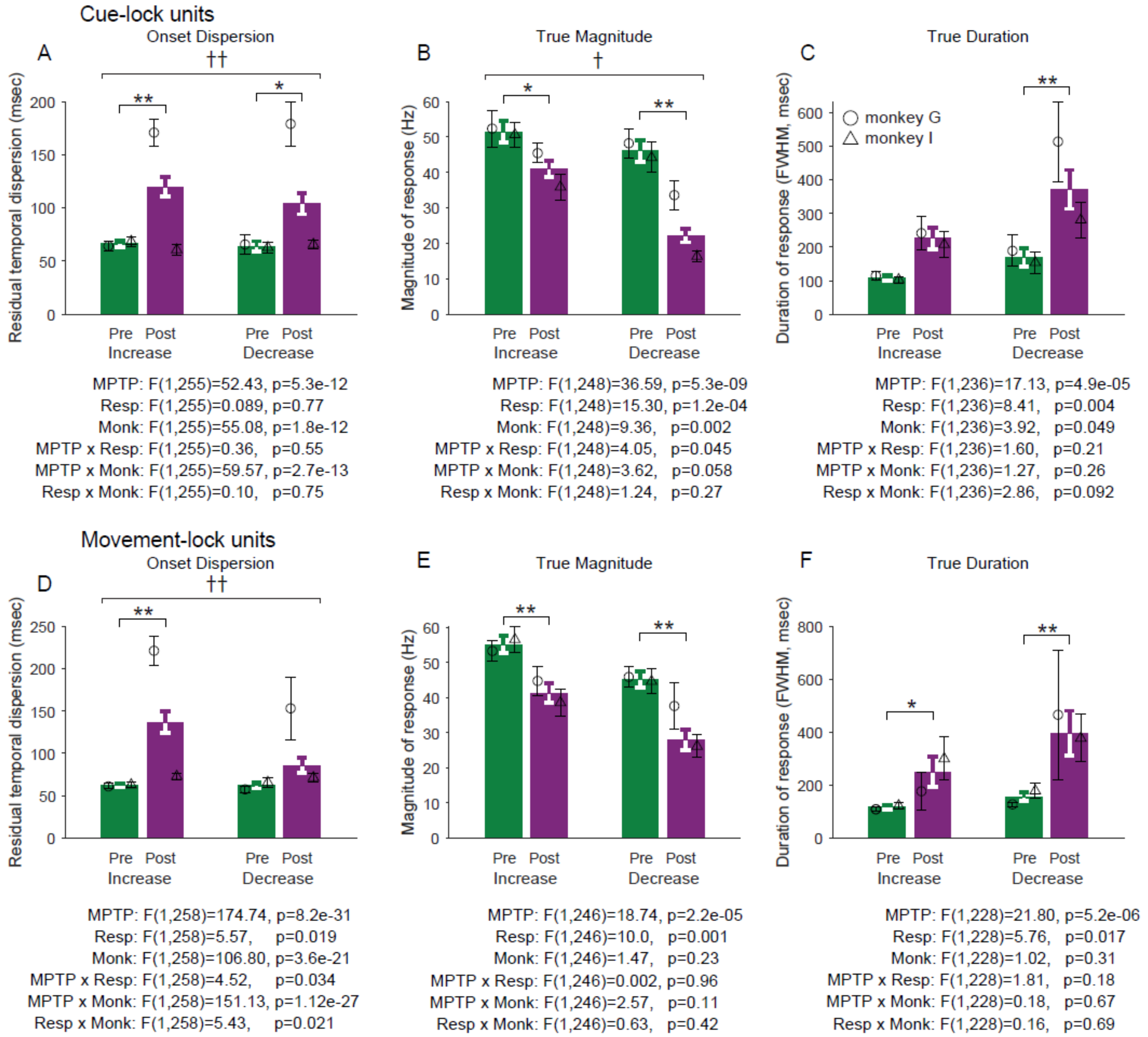

Effects of MPTP on jitter-corrected response metrics (dispersion, magnitude and duration; i.e., Fig. 6A-C) were similar in cue-locked (panels *A-C*) and movement-locked (panels *D-F*) response subtypes. Results from 3-way ANOVA (MPTP × response type × animal) are shown below.

bioRxiv preprint doi: https://doi.org/10.1101/2024.08.29.610310; this version posted May 30, 2025. The copyright holder for this preprint (which was not certified by peer review) is the author/funder, who has granted bioRxiv a license to display the preprint in perpetuity. It is made available under aCC-BY 4.0 International license.

Figure S7

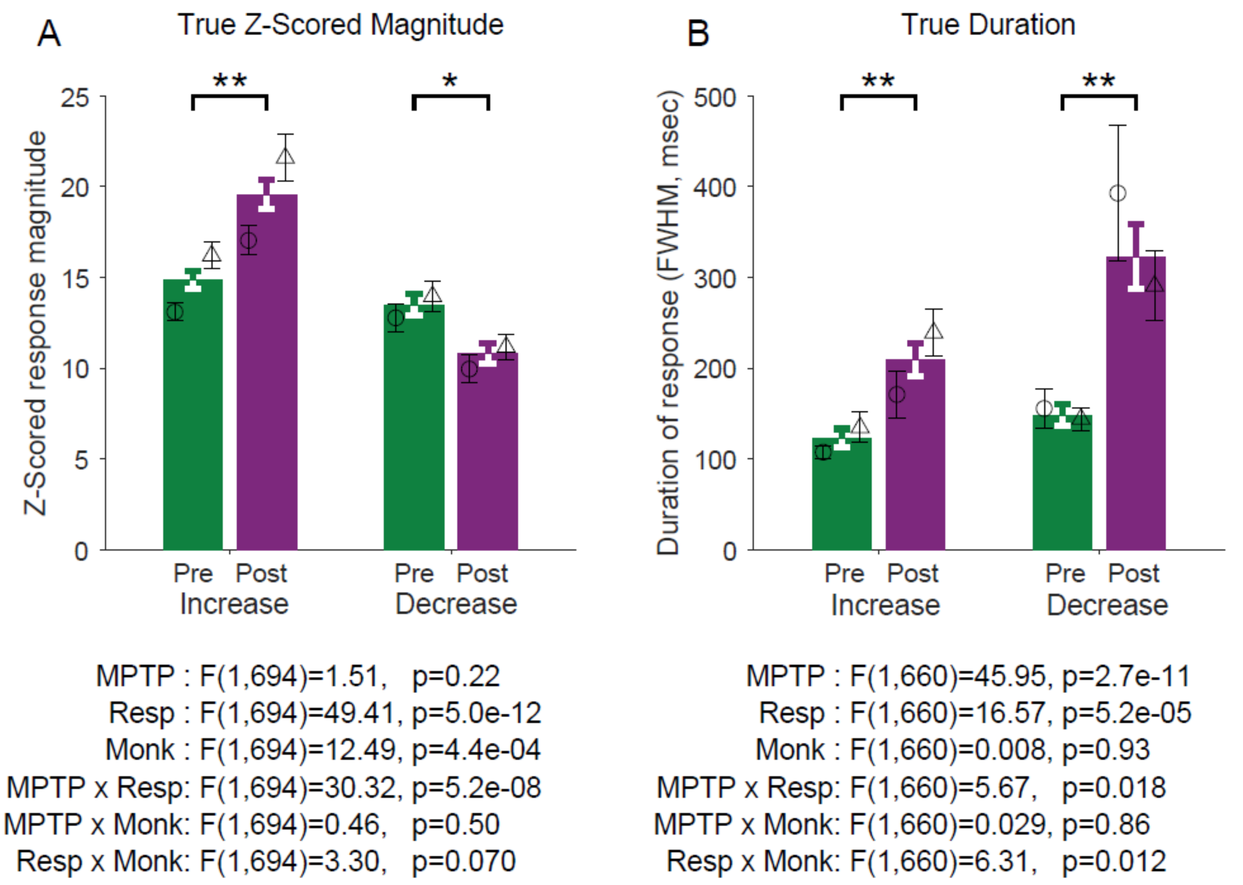

Effects of MPTP on Z-scored, jitter-corrected response metrics. *A*: The magnitude of increase-type responses was larger post-MPTP whereas the magnitude of decrease-type responses was diminished (* p<0.05, ** p<0.01, Tukey's test). *B*: Response durations were prolonged following MPTP administration (** p<0.01, Tukey's test). Results from 3-way ANOVA (MPTP × response type × animal) are shown below.
