# Supplementary material for: Movement-related activity in the internal globus pallidus of the parkinsonian macaque
Source: bioRxiv. 2024 Aug 30:2024.08.29.610310. Preprint. [Version 1] doi: 10.1101/2024.08.29.610310 (PMC11383679; doi:10.1101/2024.08.29.610310)
Supplement: Supplementary file 1 [file media-1.pdf]

Movement-related activity in the internal globus pallidus of the parkinsonian macaque

Daisuke Kase[1,2], Andrew J. Zimnik[3,4], Yan Han[1], Devin R. Harsch[1,2], Sarah Bacha[1], Karin M. Cox[1], Andreea C. Bostan[1,2], R. Mark Richardson[5], Robert S. Turner[1,2]

1. *Department of Neurobiology, Center for Neuroscience and The Center for the Neural Basis of Cognition, University of Pittsburgh, Pittsburgh, PA, USA*
2. *Aligning Science Across Parkinson's (ASAP) Collaborative Research Network, Chevy Chase, MD, USA*
3. *Department of Neuroscience, Columbia University Medical Center, New York, NY*
4. *Zuckerman Institute, Columbia University, New York, NY, USA*
5. *Department of Neurosurgery, Massachusetts General Hospital and Harvard Medical School, Boston, MA, USA*

ORCIDs: DK 0000-0001-6787-5862, DRH 0000-0001-6267-2871, KMC 0000-0002-5418-0508, ACB 0000-0003-4376-3836, RST 0000-0002-6074-4365

Address correspondence to:

Robert S Turner, PhD
Department of Neurobiology
University of Pittsburgh
4047 BST-3, 3501 Fifth Avenue
Pittsburgh, PA 15261

Phone: 412-383-5395
Fax: 412-383-9061
email: rturner@pitt.edu

SUPPLEMENTARY FIGURES

Supplementary Figure S1

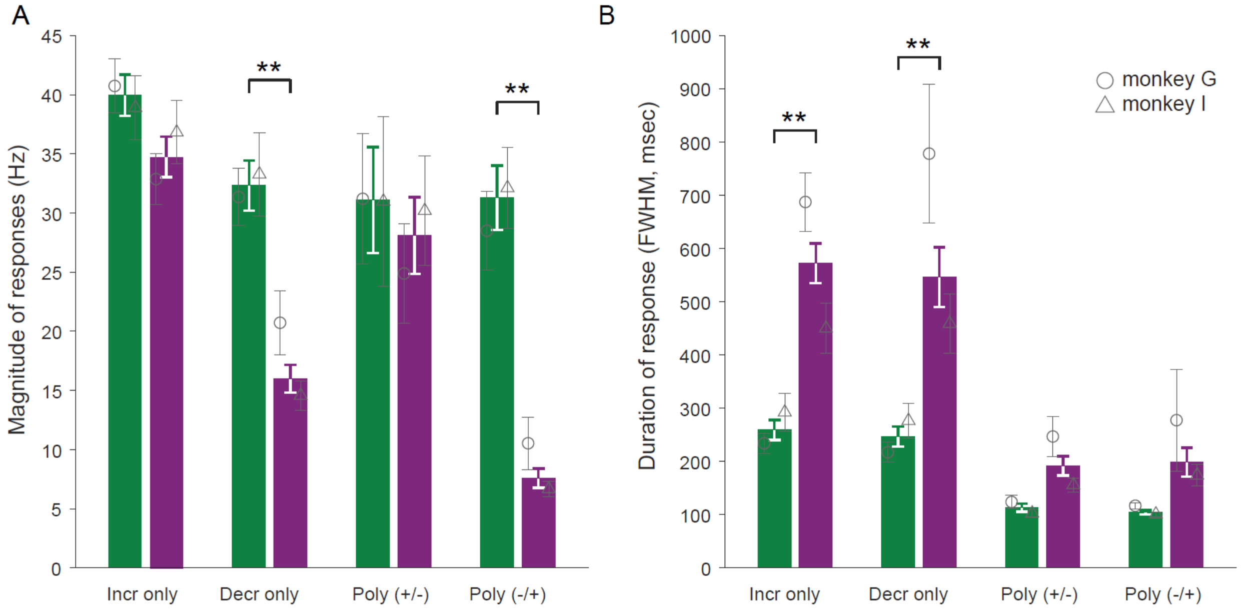

A. Response magnitudes pre-MPTP (green) and post-MPTP (magenta). Magnitudes were reduced in size following MPTP administration ($p<0.01$, 2-way ANOVA main effect of MPTP). The reduction in response magnitude was more severe in decrease-type responses (** $p<0.01$, Tukey's test). B. Response durations pre-MPTP (green) and post-MPTP (magenta). The durations were prolonged following MPTP administration ($p<0.01$, 2-way ANOVA main effect of MPTP). The prolongation of response durations was more prominent for monophasic responses (** $p<0.01$, Tukey's test).

Supplementary Figure S2

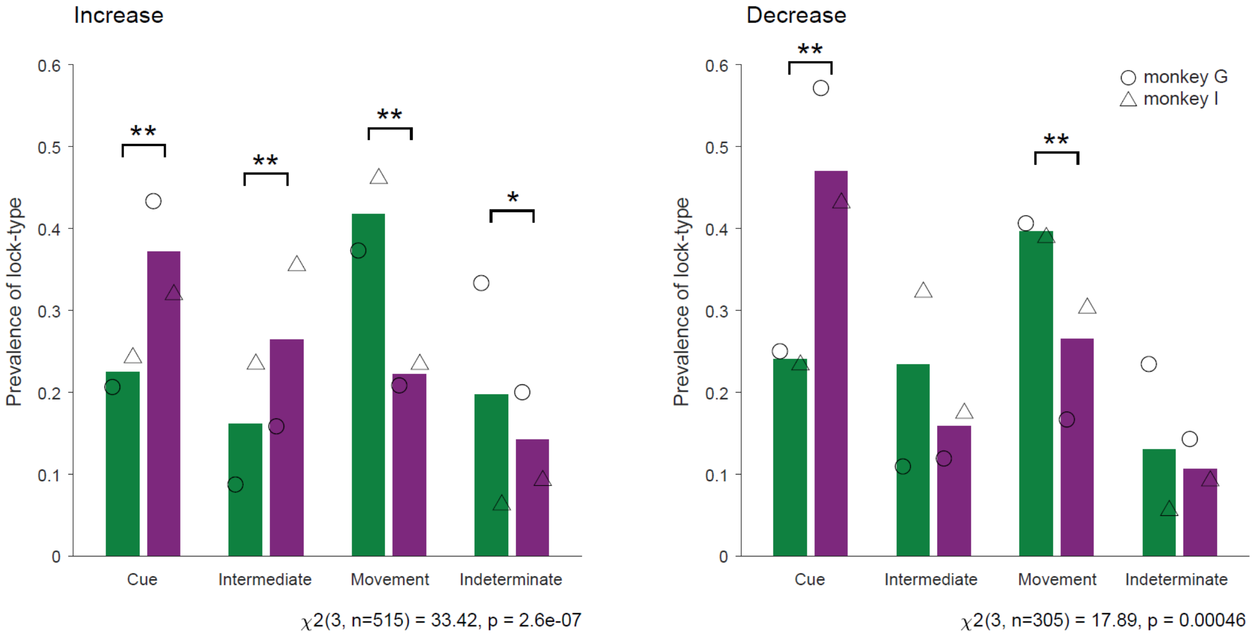

The changes in prevalence of event locking types between pre- (green) and post-MPTP (magenta) for increase- (left) and decrease-type responses (right). The peak prevalence shifted from move-locked to cue-locked responses following MPTP administration in both response types (* $p<0.05$, ** $p<0.01$, adjusted residual analysis). Open circle: animal G, open triangle: animal I

Supplementary Figure S3

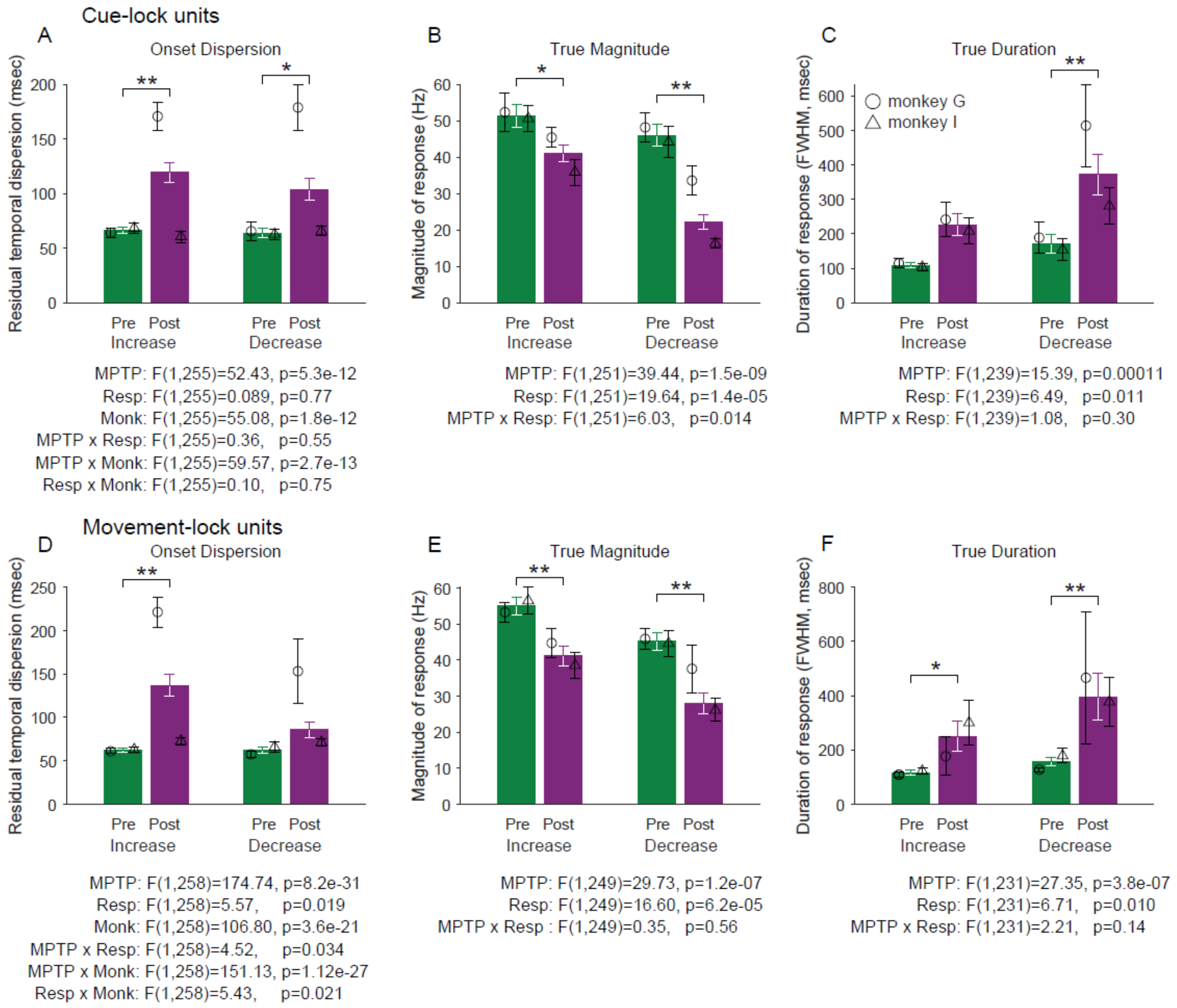

Summaries of residual temporal dispersion for Cue-locked (A) and Move-locked units (D), response magnitude from response-aligned spike-density function for Cue-locked (B) and Move-locked units (E), and response duration from response-aligned spike-density function for Cue-locked (C) and Move-locked units (F), pre- (green) and post-MPTP (magenta). Statistical differences were computed using the 3-way ANOVA for the residual temporal dispersion and 2-way ANOVA for the response magnitude and duration followed by Tukey's test; (* $p<0.05$, ** $p<0.01$). All values are presented as mean ± SEM. Open circle: animal G, open triangle: animal I.
